# Large-Scale Deep Learning–Enabled Infodemiological Analysis of Substance Use Patterns on Social Media: Insights From the COVID-19 Pandemic

**DOI:** 10.2196/59076

**Published:** 2025-04-17

**Authors:** Julina Maharjan, Jianfeng Zhu, Jennifer King, NhatHai Phan, Deric Kenne, Ruoming Jin

**Affiliations:** 1 Department of Computer Science Kent State University Kent, OH United States; 2 Department of Public Health Kent State University Kent, OH United States; 3 Data Science Department New Jersey Institute of Technology Newark, NJ United States

**Keywords:** substance use, social media, deep learning, Robustly Optimized Bidirectional Encoder Representations from Transformers Pretraining Approach, human-in-the-loop, COVID-19

## Abstract

**Background:**

The COVID-19 pandemic intensified the challenges associated with mental health and substance use (SU), with societal and economic upheavals leading to heightened stress and increased reliance on drugs as a coping mechanism. Centers for Disease Control and Prevention data from June 2020 showed that 13% of Americans used substances more frequently due to pandemic-related stress, accompanied by an 18% rise in drug overdoses early in the year. Simultaneously, a significant increase in social media engagement provided unique insights into these trends. Our study analyzed social media data from January 2019 to December 2021 to identify changes in SU patterns across the pandemic timeline, aiming to inform effective public health interventions.

**Objective:**

This study aims to analyze SU from large-scale social media data during the COVID-19 pandemic, including the prepandemic and postpandemic periods as baseline and consequence periods. The objective was to examine the patterns related to a broader spectrum of drug types with underlying themes, aiming to provide a more comprehensive understanding of SU trends during the COVID-19 pandemic.

**Methods:**

We leveraged a deep learning model, Robustly Optimized Bidirectional Encoder Representations from Transformers Pretraining Approach (RoBERTa), to analyze 1.13 billion Twitter (subsequently rebranded X) posts from January 2019 to December 2021, aiming to identify SU posts. The model’s performance was enhanced by a human-in-the-loop strategy that subsequently enriched the annotated data used during the fine-tuning phase. To gain insights into SU trends over the study period, we applied a range of statistical techniques, including trend analysis, k-means clustering, topic modeling, and thematic analysis. In addition, we integrated the system into a real-time application designed for monitoring and preventing SU within specific geographic locations.

**Results:**

Our research identified 9 million SU posts in the studied period. Compared to 2019 and 2021, the most substantial display of SU-related posts occurred in 2020, with a sharp 21% increase within 3 days of the global COVID-19 pandemic declaration. Alcohol and cannabinoids remained the most discussed substances throughout the research period. The pandemic particularly influenced the rise in nonillicit substances, such as alcohol, prescription medication, and cannabinoids. In addition, thematic analysis highlighted COVID-19, mental health, and economic stress as the leading issues that contributed to the influx of substance-related posts during the study period.

**Conclusions:**

This study demonstrates the potential of leveraging social media data for real-time detection of SU trends during global crises. By uncovering how factors such as mental health and economic stress drive SU spikes, particularly in alcohol and prescription medication, we offer crucial insights for public health strategies. Our approach paves the way for proactive, data-driven interventions that will help mitigate the impact of future crises on vulnerable populations.

## Introduction

### Overview

Substance use (SU) is a pressing public health issue in the United States, with 58.7% of Americans aged ≥12 years using tobacco, alcohol, or illicit drugs in 2020, with an annual increase of 3.8% [1]. This includes 50% alcohol users, 18.7% tobacco users, and 13.5% illicit drug users [1]. The consequences of SU, such as deteriorating health and increased crime, have led to a significant rise in drug overdose deaths, reaching >91,000 in 2020 and >106,000 in 2021 [2]. The economic cost is substantial, with an estimated US $249 billion for alcohol misuse and >US $193 billion for illicit drug use annually [3]. The financial and health repercussions of SU demand a strategic focus on prevention research. Allocating resources to explore and counteract the causes of drug use can lead us toward a healthier and more economically resilient society.

### Background

The year 2020, commonly referred to as the COVID-19 year, holds historical significance for health care researchers due to the emergence of the deadly coronavirus. The COVID-19 pandemic exhibited a profound connection with preexisting SU and mental health issues [4-6]. Various consequences, such as economic instability, social isolation, bereavement, and restricted access to health care services, escalated anxiety and stress levels among the population [7-10]. According to the Centers for Disease Control and Prevention, data as of June 2020 revealed that 13% of Americans reported initiating or intensifying SU as a means of coping with stress or emotions related to COVID-19 [11]. The Overdose Detection Mapping Application Program reports indicated an 18% rise in drug overdoses in the early months of the pandemic compared to the same period in 2019 [12]. Several other studies [13-15] also highlighted that changes in drug availability contributed to a rise in deaths related to illicit opioid use; for instance, if heroin became less accessible, individuals might resort to the more potent fentanyl.

Simultaneously, the COVID-19 pandemic led to internet use of up to 70% [16], leading to a record 11.1% growth in Twitter’s (subsequently rebranded X) user base in 2020. This surge in social media engagement, while providing a vital connection for many, has also been directly linked to an increase in SU [17]. Research studies [18-22] have indicated the negative impacts of social media on mental health, including increased anxiety, depressive symptoms, and psychological burdens related to COVID-19, which have been correspondingly linked to an increase in SU as individuals seek coping mechanisms. Notably, previous studies [23-27] have also shown a strong correlation between social media use and SU, with evidence of users being influenced to use substances by their peers’ behavior, such as tagging their social connections in their posts [26,27]. A few research studies [23,25] provide evidence that higher levels of exposure to substance-related content tend to develop positive norms and attitudes toward alcohol and drug use. Likewise, a study also showed that adolescents who are regularly active on social media have a greater likelihood of subsequent tobacco or cannabis use initiation [24]. In our research, we aim to identify these gaps in the knowledge of SU during the COVID-19 pandemic by analyzing social media content and making a comparison with pre- and postpandemic years. We achieve this through a deep learning model alongside various statistical methods. By comprehending the findings, the ultimate goal is to support public health sectors to develop more effective prevention and intervention strategies to control and prevent SU during global crises.

### Related Studies

The onset of the COVID-19 pandemic has notably intensified global research on drug crises. Numerous studies [6,7,28-50] have examined the intersection of drug use and the pandemic’s societal impacts. These investigations commonly revealed a significant correlation between the pandemic and shifts in SU patterns, impacting both people with or without SU disorder (SUD). Various studies [6,28,36,37] evidenced that the disruption in health care services during the COVID-19 pandemic period primarily impacted people with SUD and was thus linked to higher abuse of substances. However, many of those research studies relied on data from small cohorts [18,22,29,30] that predominantly used methodologies such as surveys or interviews for data collection. Few studies [30,35,39-41,47,48,51] have used social media data to explore SU during the pandemic. However, the scope of such studies often remains limited to peak pandemic periods and typically focuses on specific types of drugs, such as alcohol, tobacco, or opioids. Only 2 of the studies [14,52] accounted for multiple drug types (that are mostly consumed) to study the correlation between COVID-19 and use of substances, but they still did not consider other drug types (that are less widely used) to check if the use was altered during the global crisis. Likewise, most research only accounted for the peak pandemic period to study the SU trend during COVID-19. Only the study by Omare et al [47] accounted for the prepandemic period (2016-2020) as the baseline to compare the SU trend before the COVID-19 pandemic. Essentially, it established 2 prepandemic baselines, that is, 2016 to 2018 and 2018 to 2019, and compared SU trends over the studied period. However, it did not account for the postpandemic period or whether the SU was altered due to the consequences of COVID-19. This highlights a gap in the literature, underscoring the need for more expansive research that covers various substance types and multiple time frames to better understand the long-term impacts of the pandemic on drug use patterns.

Prominent national agencies such as the National Survey on Drug Use and Health [1], the National Institute on Drug Abuse (NIDA) [Centers for Disease Control and Prevention [2], and the Substance Abuse and Mental Health Services Administration [50] routinely perform national-level analyses of drug use. Traditionally, these reports are based on survey methodologies, which may involve a relatively limited participant pool. The COVID-19 pandemic further complicated these efforts, limiting face-to-face data collection and necessitating a shift toward online surveys. This change compromised the depth and reliability of data in 2020; for example, the 2020 National Survey on Drug Use and Health report only includes data from the first quarter and used web-based methods for the fourth quarter [1]. In addition, the transition from *The Diagnostic and Statistical Manual of Mental Disorders, Fourth Edition* to *The Diagnostic and Statistical Manual of Mental Disorders, Fifth Edition* during this period introduced challenges in comparing the new data with those from previous years due to methodological changes.

The existing studies were limited to fewer drug types and demographics of smaller cohorts that mainly focused on the peak pandemic period and did not account for trends before and after the pandemic. Thus, in our research, we have aimed to use large-scale social media data to examine a broader spectrum of drug types, aiming to provide a more comprehensive understanding of drug use trends during the COVID-19 pandemic.

Previous research on social media often used keyword-based and traditional machine learning approaches to analyze drug-related content. Notably, studies [52,53] have identified potential SU incidents using keyword-based methods, that is, by detecting specific drug names such as Adderall, oxycodone, quetiapine, metformin, cocaine, marijuana, weed, methamphetamine, tranquilizer, etc. However, these keyword-based methods are limited, as they often fail to discern the context in which terms are used, resulting in significant ambiguities [54]. Users frequently use slang and metaphorical language that these models cannot adequately interpret. In addition, other studies [53,55-57] have used traditional machine learning classifiers such as naive Bayes, support vector machines, and decision trees. While enhancements such as word2vec for word embedding have been applied, these methods typically struggle with the subtleties of language used in social media. Despite some advancements in sequence-based models, such as long short-term memory or convolutional neural networks [54,58], these approaches still fall short of fully understanding contextual meanings, a challenge effectively addressed by the attention mechanism [59]. Thus, in our research, we have adopted the Robustly Optimized Bidirectional Encoder Representations from Transformers Pretraining Approach (RoBERTa) [60] model, which leverages an advanced attention mechanism to overcome these limitations. This implementation represents a novel application in the analysis of large-scale social media data for drug use studies. Despite the challenges posed by limited annotated data availability, we have incorporated an iterative learning process inspired by human-in-the-loop (HITL) [61] and active learning techniques [62] to further enhance the accuracy of our model. This approach not only refines the model with each iteration but also focuses on learning from the most informative data points, streamlining the data annotation process.

In summary, in this research, we sought to study a large amount of data from Twitter spanning a 3-year period, including the prepandemic (2019) and postpandemic (2020) periods as baseline and consequence periods, to identify the patterns of drug use using a deep learning model (RoBERTa) and various other statistical methods (trend analysis, k-means clustering, topic analysis, and thematic analysis), which are explained in the Methods section in detail. In addition to this, we also aim to analyze different types of drugs and themes in the SU discourse. Specifically, we aim to answer the research questions presented in Textbox 1.

**Research questions.**

1. How did the discourse on substance use (SU) evolve on Twitter (subsequently rebranded X) from 2019 to 2021, and what variations existed in the distribution of different substances during this time?
2. Following the announcement of the pandemic, what were the primary substance types that garnered significant discussion, and what were the themes of these dialogues?
3. How did the prevalence of the studied theme influence various types of substances during the underlying study period?
4. How did the identified themes correlate with the substance types?
5. What primary discussion topics arise from k-means analysis, specifically during the study period?
6. To what degree does the classifier’s effectiveness in pinpointing SU-related tweets during the pandemic align with or differ from GPT-3?
7. How has the overall system contributed to the real-time tracking of SU, as evidenced by the research?

### Contributions

The study’s main contributions are as follows:

1. A large-scale SU behavior tweet collection system with expert-annotated tweets for supervised learning
2. A customized pretrained language model based on social media data (Twitter) and an iterative supervised deep learning algorithm for detecting SU posts
3. Insightful statistical analysis of the identified SU posts
4. A real-time search engine–based application for monitoring SU in temporal and spatial dimensions

## Methods

**Figure 1:**
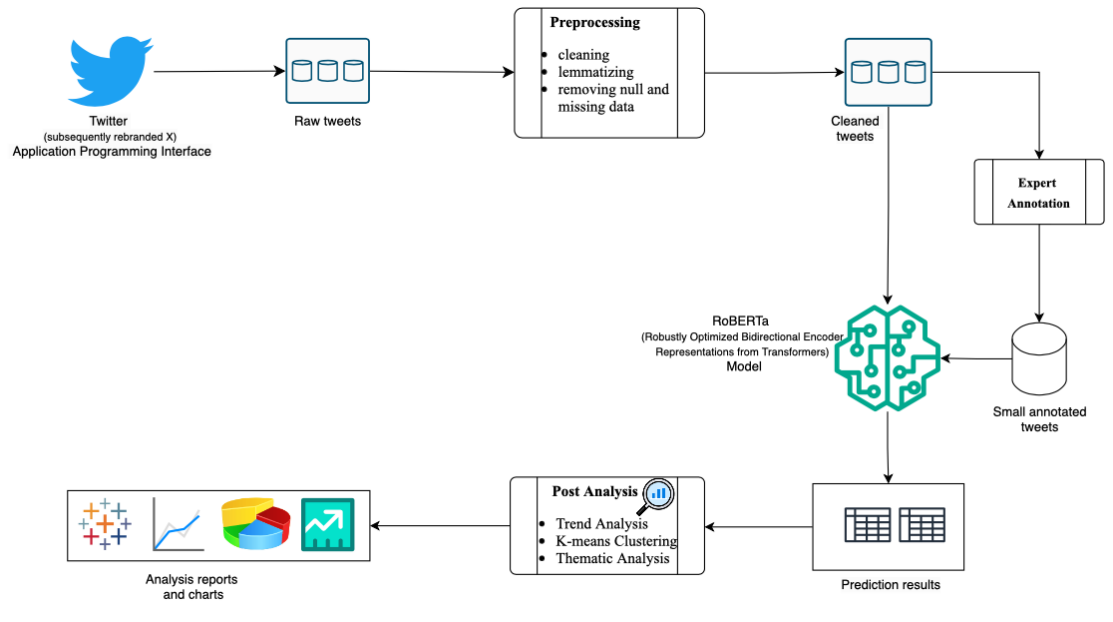
Comprehensive research overview flowchart. API: application programming interface; BERT: Bidirectional Encoder Representations from Transformers; NA: not available; RoBERTa: Robustly Optimized Bidirectional Encoder Representations from Transformers Pretraining Approach.

Figure 1 shows the overall methodology used in the research. All the steps mentioned in the flow diagram are described subsequently.

### Data Collection

For this research, historical Twitter data were obtained from the Internet Archive [63], a digital library committed to providing free access to a wide array of digital information, including web pages, texts, audio, and videos. This nonprofit organization archives digital content to preserve it and make it accessible for future research. Among its many resources, the Internet Archive includes collections of Twitter data, which consist of tweets captured until July 2023. In our research, we downloaded the raw tweet data covering the period from January 2019 to December 2021. Initially, the data downloaded from this source were in compressed JSON formats, consisting of a large set of files for each day. A pipeline script was developed to extract these files and consolidate them into single-day JSON files. During the extraction process, we retrieved only the time stamp and the actual text of the posts for our analysis. It is important to note that the raw tweets for some days were missing in the data source, specifically in February 2020, January 2021, and April 2021. This absence resulted in skewed time series plots in these months, as discussed in the Results section.

### Data Preprocessing

The preprocessing of raw tweets was a crucial initial step to ensure the quality and relevance of the data for further analysis. To efficiently preprocess the large-size files, we divided each daily JSON file into smaller chunks, loaded them in memory, processed each chunk individually, and then merged them back into a single file. The preprocessing steps are described subsequently.

**Figure 2:**
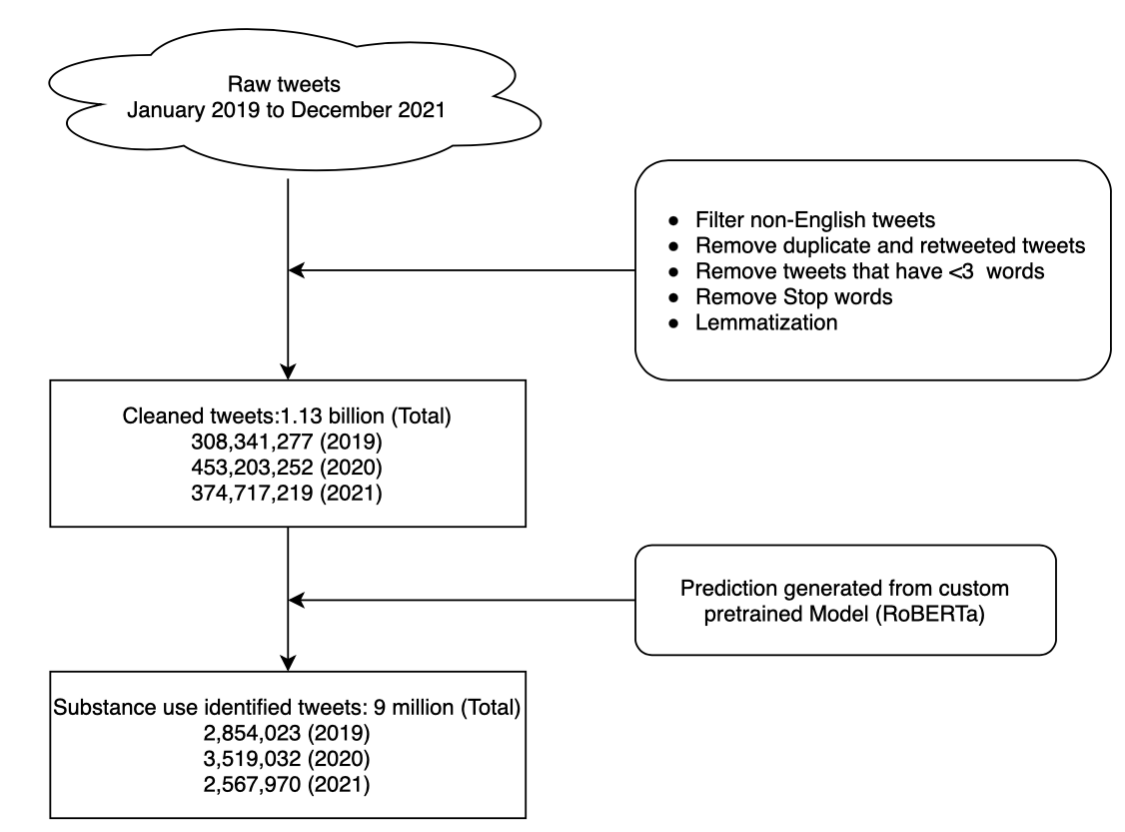
Flowchart of tweet processing. RoBERTa: Robustly Optimized Bidirectional Encoder Representations from Transformers Pretraining Approach.

Initially, we filtered out all non-US tweets and duplicate or retweeted tweets to focus our research on English-language tweet posts and reduce redundancy, respectively. Then, we cleaned the text data by removing punctuation and stop words using the Nature Language ToolKit (*NLTK*) package and converted all characters to lowercase to maintain uniformity and prevent discrepancies caused by case sensitivity. Subsequently, we also replaced all the usernames, URLs, and hashtags in the post with the keywords “USER,” “HTTPURL,” and “HASHTAG” to hide the users’ identity and ease semantic understanding. Then, we performed lemmatization using the *NLTK* package to reduce words to their base form (eg, “drinking” to “drink”) to standardize text and improve consistency. Finally, we removed tweets containing <3 words, as these were deemed too brief to provide substantive insights. This comprehensive preprocessing approach resulted in a refined dataset of 1.13 billion cleaned tweets (n=308,341,277, 26.84% in 2019; n=453,203,252, 40.05% in 2020; and n=374,717,219, 33.11% in 2021) poised for further analysis, as depicted in Figure 2.

### Feature Extraction and Data Annotation

#### Overview

Three specialized domain experts in mental health, SU, and public health performed the data annotation process. The main purpose of annotating data was to serve as a seeding dataset to train our deep learning RoBERTa model. Furthermore, as we intended to identify the SU posts in natural language social media data (where users might not clearly mention the drugs but still talk about SU), our goal was to collect and annotate data that were based on context rather than just keyword-based posts. Thus, we first outlined the context of SU based on 3 main criteria, namely *Types of Substance*, *Uses of Substances*, and *Intent to Use a Substance*.

#### Types of Substance

Substance type posts usually indicated either direct mention of drug names (that could be slang or street names) or described consuming them with or without actual drug names by specifying slang. The detailed list of such drug names, along with the street names and slang, are outlined in Table S1 in Multimedia Appendix 1. For instance, the tweet, “Man, just chill and smoke weed” had a direct mention of the substance “weed” with a clear meaning of SU. Likewise, “Just smoked a joint after work” had an indication of cannabis use hinted by the keyword *joint* even though the post had not specified the actual drug name. We acknowledge that Table S1 in Multimedia Appendix 1 contains a wide range of keywords or slang that might not have a direct association with SU. Hence, careful consideration has been made while annotating posts that contain slang but do not refer to SU. One counterexample of this would be “His joints and bones ache and his muscles seize up.” Here, the post does not have any context with SU even though it contains the keyword *joint*. Hence, it is labeled as a non-SU post.

#### Uses of Substances

SU posts were identified as posts that described the context of the use of substances, including experiences, effects, or consequences of consumption. The description usually covered personal anecdotes, stories, testimonials, promotions, advice, or recommendations about consumption, and information on obtaining substances. Examples included posts such as, “Feeling relaxed and happy after taking my meds—Xanax does wonders” and “Anyone needs advice on chilling out? I swear by CBD gummies.” In both examples, the post specified the consequence of consuming substances without mentioning the actual names of the substances.

#### Intent to Use a Substance

Substance intent posts were posts that exhibited actions or behaviors suggesting preparation for specific plans to engage in or a desire for SU and were classified as indicative of SU. Examples included “Planning to get some crystal tonight, can’t wait” and “Thinking about getting high this weekend to unwind.” These examples indicated the actual plan of consuming the substance without clearly mentioning the substance type.

Once the context of SU had been outlined, we proceeded with collecting tweets to annotate. We collected a subset of raw tweets (ie, without cleaning or preprocessing) from January 2020 through April 2020 and asked each domain expert to independently review and annotate a batch of collected tweets under the previously defined criteria. The annotation for each single post was confirmed only if at least 2 annotators voted the same. Upon discrepancies, the annotators further convened to discuss and repeated the process until a consensus was met. This iterative process ensured high reliability and validity in identifying instances of SU. The final annotation resulted in a corpus of 4011 posts. Sample examples of annotated SU and non-SU tweets are included in Table S2 in Multimedia Appendix 1. This thorough annotation process aided in creating a reliable training dataset for fine-tuning our SU classifier.

### RoBERTa Model for Tweet Classification

#### Overview

In our research, we used the RoBERTa model [60], an advanced iteration of the Bidirectional Encoder Representations from Transformers (BERT) model [64], which itself marked a significant breakthrough in natural language processing. Developed by Google, BERT harnesses the power of the transformer architecture [59], notable for its innovative attention mechanism. This mechanism generates word embedding that captures deep contextual meanings within the text by enabling the model to consider each word in the context of all other words in a sentence rather than in isolation. The BERT model is structured to undergo 2 training phases, a pretraining phase and a fine-tuning phase, which is advantageous when adapting to specific tasks with limited available data. During the pretraining phase, the model learns general language patterns from a large text corpus through the masked language model (MLM) and next sentence prediction (NSP). MLM encourages the model to predict missing words based only on their context, enhancing its understanding of language nuances. NSP trains the model to understand the relationships between consecutive sentences, which is vital for tasks that require an appreciation of text flow. The fine-tuning phase then specifically adapts the pretrained model to nuanced tasks using smaller, specialized datasets, ensuring that the model maintains robust performance by refining the comprehensive linguistic capabilities developed during pretraining. Our objective could have been achieved by the BERT model; however, the elimination of the NSP task in the RoBERTa model simplifies the architecture, thereby making it the best fit for our use case. Unlike in BERT, RoBERTa only focuses on capturing contextual meaning (on the MLM task) rather than sentence relationships (on the NSP task), which is more relevant to tweet dataset context because tweet data are usually short sentences that do not require sentence relationship information. This modification makes RoBERTa more robust without compromising all the key features of BERT.

**Figure 3:**
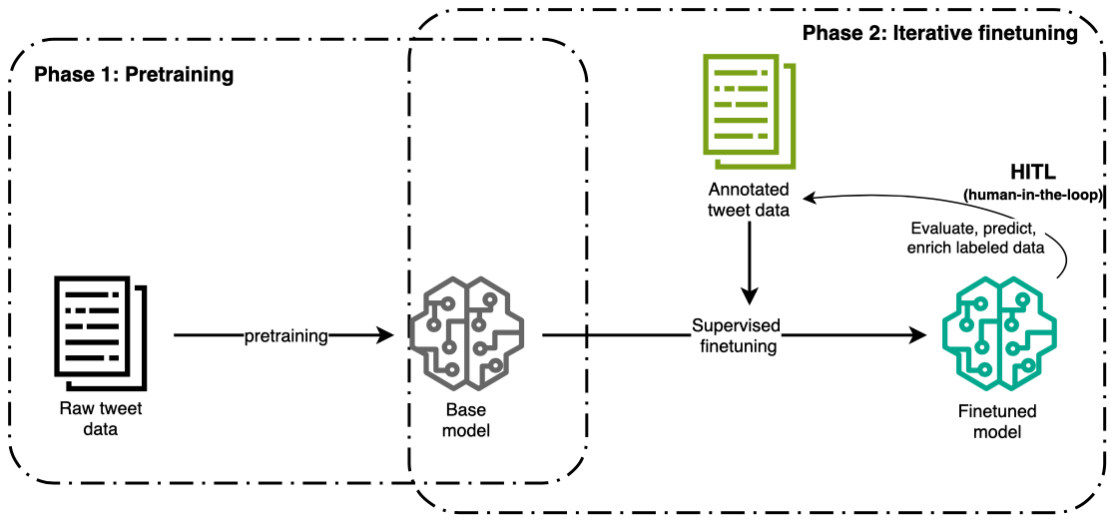
Illustration of training 2 phases Robustly Optimized Bidirectional Encoder Representations from Transformers Pretraining Approach model for substance use tweet classification. HITL: human-in-the-loop.

In the subsequent subsections, we explain the pretraining and fine-tuning phases carried out in our research, as depicted in Figure 3.

#### Pretraining From Scratch

The specific linguistic challenges of our Twitter dataset necessitated a training-from-scratch approach to avoid biases from generic training datasets. In our method, we primarily adopted this customized approach to learn the language understanding of social media data from 33 million raw Tweet posts as shown in the phase 1 of Figure 3.

Initially, we performed tokenization using ByteLevelBPETokenizer. Essentially, this sub–word-level tokenizer broke down words into subword units, allowing it to handle out-of-vocabulary words and rare words more effectively than word-level tokenizers, thus enabling more coverages and generalizations in domains with specialized terminology such as ours. We used 8192 vocab_size and min_frequency 2 as hyperparameters, along with [“<s>”, “<pad>”, “</s>,” “<unk>”, “mask>”] special tokens to indicate the start of the sentence token, padded token, end of the sentence token, unknown token, and masked token, respectively. Additional parameters are provided in Tables S3 and S4 in Multimedia Appendix 1.

After tokenization, we split the original dataset into 2 splits: as the training set (n=29.7 million, 90%), the testing set and (n=3.3 million, 10%). Then, we started training with the MLM objective, where a fraction of tokens (n=4.45 million, 0.15%) in each input sequence were masked, and the model learned to predict them based on contextual information. Training proceeded iteratively using stochastic gradient descent, with hyperparameters tuned based on validation performance. The model achieved a perplexity of 3.84 on the test data, which served as a baseline evaluation of the model. We ensured our language model was efficient by further evaluation after the fine-tuning step.

#### Iterative Fine-Tuning

##### Overview

After our model successfully deciphered language understanding in social media data, our next objective was to leverage this knowledge to distinguish posts related to SU. We achieved this by incorporating an additional binary classification layer into the existing model and retraining it with a newly labeled dataset, as depicted in the phase 2 of Figure 3. As with the unsupervised pretraining, we divided the dataset into training, validation, and test splits. We then retrained (fine-tuned) the newly annotated dataset, adjusting the model’s weights by calculating the error between the predictions and the actual labels using an optimization algorithm.

However, to prevent overfitting due to the limited size of the initially labeled data, we adopted an iterative fine-tuning approach inspired by HITL [61]. HITL is a collaborative technique that integrates human input at various stages of model development, such as training, testing, feedback, and decision-making [61]. In our case, we used HITL in only the training phase. We used human reviewers to only assess the model’s prediction results, which were then used to further train the model in successive rounds. Specifically, the process began with training the model on a seed-labeled dataset, followed by generating predictions for unseen data. Furthermore, these predictions were reviewed by human experts to refine and enrich the annotated dataset, which was used for subsequent training. This iterative cycle of training, prediction, and human review continuously improved the model’s performance by enhancing the quality of the training data.

The overall steps in our fine-tuning phase are detailed in the algorithm mentioned subsequently.

##### Step 1: Data Split

Before training the model, we split our 4011 annotated dataset into 3 sets: the training set (n=3208, 80%), the testing set (n=402, 10%), and the validation set (n=401, 10%).

##### Step 2: Initial Fine-Tuning and Cross-Validation

The initial parameters from the pretrained model were initialized. Then, the model was fine-tuned with the training dataset for 32 epochs on a batch size of 16 and a learning rate of 2e-5. A dropout layer was added to prevent overfitting, and the model was evaluated using a separate held-out dataset to ensure unbiased parameter tuning.

##### Step 3: HITL for Generating a New Labeled Dataset

###### Overview

Our ultimate objective at this step was to leverage human experts to pinpoint crucial data points that could enrich the annotated dataset and refine the model’s accuracy. Human experts reviewed the model’s predictions on unseen data and then identified and corrected errors. This feedback (corrected data) was then used to train the model in the next iteration.

###### Step 3.1: Prediction of Unseen Data

We used the refined model from step 2 to generate predictions of new, unseen data.

###### Step 3.2: Expert Review on Positive Predictions

**Figure 4:**
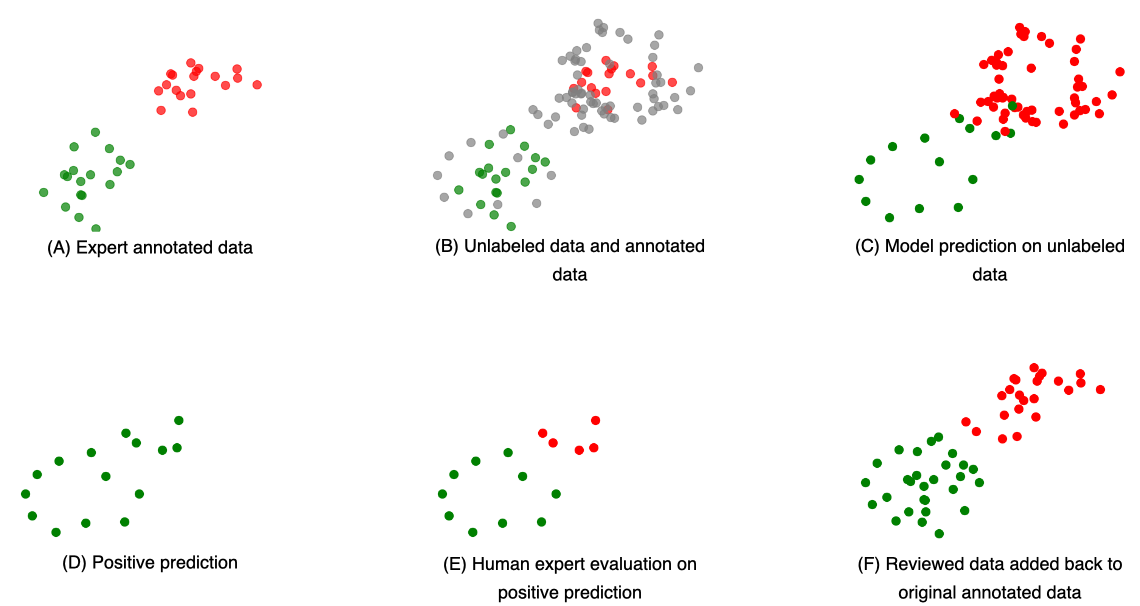
Illustration of datasets in different stages used in the iterative fine-tuning phase. SU: substance use.

Due to the severe imbalance in the dataset (0.05 positive, 0.95 negative), as shown in Figure 4, we focused exclusively on reviewing positive predictions. Concentrating on positives and making corrections to false negatives allowed us to directly improve the model’s sensitivity and precision. This approach ensured that the model better recognized the critical but infrequent true positive cases, thus enhancing overall accuracy and robustness.

###### Step 3.3: Annotation

We leveraged our expert knowledge to annotate misclassified positives (false negatives) as negatives and correctly identified positives (true positives) as positives.

###### Step 3.4: Bias Reduction

To address potential bias by specifically focusing on positive predictions, we selectively reviewed a subset of complex positive predictions, which were likely to be misclassified as negatives. For example, posts using metaphoric language or slang, such as “riding the white horse,” to indicate heroin use, required nuanced interpretation beyond simple keywords. By targeting these complex positives, we aimed to reduce the bias of not reviewing negative predictions. This careful attention to positive predictions ensured that we minimized the risk of failing to identify true instances of SU hidden within the data labeled as negative.

###### Step 3.5: Outcome

The final subset of true positives from Bias Reduction and misclassified positives from Annotation were considered as newly annotated data. In each iteration, we made sure the outcome contained 100 positive and 100 negative posts.

##### Step 4: Expansion of the Original Annotated Dataset

The outcomes from step 3 were added to the original annotated dataset.

##### Step 5: Evaluation and Iteration (Repeating Steps 1, 2, 3, and 4)

The fine-tuning was carried out for 20 iterations, expanding the annotated dataset in each iteration up to 6400 entries. At each round, we evaluated the model’s accuracy on the test set and repeated the process until we achieved the desired accuracy of 80%.

In addition to achieving 80% accuracy, the model demonstrated strong performance across other metrics, with a recall of 79%, a precision of 85%, and an *F*_1_-score of 81%. These scores indicated that the classifier effectively identified most SU instances while maintaining a low rate of false positives, ensuring balanced overall performance.

### Substance Definitions and Their Types

A substance encompasses any psychoactive compound that can be legal, illegal, or medically prescribed, with potential impacts on health and society, including the risk of addiction. In our study, we classified substances into 10 primary categories based on their pharmacological and behavioral effects, following the categorization provided by the NIDA [65] and the Drug Enforcement Administration [66].

These categories are presented in Textbox 2.

The specifics for each substance category, including associated keywords, are detailed in Table S1 in Multimedia Appendix 1.

**Classification of substances into 10 primary categories based on their pharmacological and behavioral effects.**

1. Tobacco: includes cigarettes, vapor cigarettes, cigars, chewing tobacco, and snuff
2. Alcohol: covers all forms of beer, wine, and distilled spirits
3. Cannabinoids: encompasses marijuana, hashish, hash oil, and edibles containing cannabinoids
4. Opioids: includes drugs such as heroin, methadone, buprenorphine, oxycodone, Vicodin, and Lortab
5. Stimulants: includes cocaine, amphetamines, methamphetamine, methylphenidate (eg, Ritalin), and atomoxetine (eg, Strattera)
6. Club drugs: includes 3,4 methylenedioxymethamphetamine (MDMA) or ecstasy and gamma hydroxybutyrate (GHB)
7. Hallucinogens: lysergic acid diethylamide (LSD), psilocybin, mescaline, and dimethyltryptamine
8. Dissociative drugs: ketamine, phenyl cyclohexyl piperidine (PCP), and dextromethorphan
9. Prescription medications: a broad category that includes antibiotics, analgesics, statins, antidepressants, antihypertensives, hormonal contraceptives, and anticoagulants
10. Other compounds: features synthetic cannabinoids (eg, K2 or spice), anabolic steroids, inhalants, and synthetic cathinone (eg, mephedrone and methylenedioxypyrovalerone [MDPV])

### Baseline Themes

The relationship between COVID-19 and SU patterns has garnered significant attention, with the COVID-19 pandemic serving as a critical case study. Previous research [4-6,14,15,31,36,37,67-70] highlighted various thematic areas that significantly influence SU, including stress and concerns related to COVID-19, economic instability, social dynamics, mental health issues, and disruptions in drug supply and health care services. Our study encompassed 6 key themes—COVID-19, economic factors, social influences, mental health, supply chain disruptions, and health care disruptions, as presented in Table 1. A short description of each theme and the impacted individuals (target population), along with study references, are listed in Table 1. To identify the themes in our dataset, we performed latent Dirichlet allocation (LDA) topic analysis to extract the tokens associated with each theme. Then, we refined the list of these tokens with the help of our experts. The complete list of tokens for each theme is detailed in Table S5 in Multimedia Appendix 1.

**Table 1:** Six major themes that impacted substance use during the global COVID-19 pandemic.

Themes	Description	Target	Studies
COVID-19	Worry or fear related to the virus and lockdown	All people with or without SUD^a^	[4,5,31,36,67]
Economic	Financial instability, job stress, housing, and food insecurity	All people with or without SUD	[5,68-70]
Social	Stress caused by the COVID-19 lockdown, social distancing policies, and change in daily routine	All people with or without SUD	[5,36,37,68,70]
Mental health	Anxiety and depression before COVID-19	Especially people with SUD	[4-6,36,37,68,70]
Supply disruption	Drug market disruptions	Especially people with SUD	[4,13-15]
Medical disruption	Decreased access to substance use treatment, harm reduction, and emergency services	Especially people with SUD	[4-6,68,70]

^a^SUD: substance use disorder.

### Trend Analysis

#### Overview

Trend analysis is the most common technique for identifying patterns over time. In our study, trend analysis involved tracking and analyzing changes in types, discussion patterns, and themes associated with identified SU posts. We mainly used substance-type trend analysis, theme trend analysis, and k-means clustering analysis. At first, we identified the substance type, themes, and discussion pattern for each post by the keyword analysis based on Tables S1 and S5 in Multimedia Appendix 1, LDA topic analysis, and k-means clustering, respectively. Then, the subsequent trend analysis was performed.

#### Substance-Type Trend Analysis

To identify the substance type in the post, we first formulated a list of street names and slang words associated with the substance and labeled it with corresponding types, such as labeling post 1 for tobacco type if it contained any terms related to tobacco substance. The samples of posts, along with the identified substance type, are presented in Table S6 in Multimedia Appendix 1. Following identification, we aggregated the posts according to type and visualized the time series and histogram plot to identify and compare the growing trends in each substance type.

#### Theme Trend Analysis via LDA Topic Modeling

Theme trend analysis is a methodological approach that combines elements of theme analysis and trend analysis to understand how specific themes or topics evolve within a dataset. In order to understand key topics of discussion, we used LDA topic modeling [71], a powerful unsupervised machine learning technique, to discover abstract topics within a collection of documents. We used this to answer question 2 (Textbox 1) specifically, where we generated the top 10 topics with the top 10 keywords and categorized the topics based on the identified baseline themes.

### k-Means Clustering Analysis

k-means clustering is an unsupervised machine learning algorithm used to partition a dataset into k distinct, nonoverlapping clusters based on the similarity of data points by minimizing the variance within each cluster and maximizing the variance between different clusters [72]. The algorithm iteratively assigns data points to one of the k clusters based on the closest mean (centroid) of the cluster until the positions of the centroids stabilize. In our case, we used the scikit-learn library to perform the k-means clustering, where we used the term frequency-inverse document frequency scheme to create vectorization and considered the elbow method to identify the value of k for performing the clustering.

### Thematic Analysis

Thematic analysis is used in qualitative research to analyze and interpret theme patterns within qualitative data. In our study, we used heat map analysis and factor analysis [73-75] to visually explore the relationship between identified themes and types of substances and to identify latent factors (patterns) from the observed themes, respectively.

### Integration in Real-Time Application

We also integrated the trained model into a real-time application to monitor SU using the Elastic Logstash Kibana stack. We set up a search engine framework—using search database [76] and logstash [77]. Elasticsearch is an open-source, distributed, RESTful, JSON-based search engine originally based on Lucene (Solr) search that stores the document or the JSON object in an inverted index structure and allows the fastest full-text search. Logstash is a server-side data processing pipeline that usually sources or sinks data to and from multiple sources. In our work, we leveraged this pipeline to ingest the document and tweets from MongoDB [78], transformed the document by adding a custom call to generate a prediction result from the trained model, and finally wrote the document in Elasticsearch. The final document was a JSON comprising a tweet body with an additional prediction field from the trained model. Meanwhile, the Elastic Logstash Kibana stack had a built-in visualization tool to generate different trending charts based on real-time data during the development phase. We developed a full-fledged application in AngularJS and ReactJS frameworks for the client’s real-time purposes. The snapshots demonstrating the chart showing the temporal and spatial analysis based on different filters are presented in the *Results* section.

### Comparison With GPT-3

The advent of large language models, particularly GPT-3 [79], seemed to have raised questions regarding the efficiency and validity of custom models such as ours. Thus, we compared the reliability of our RoBERTa model and GPT-3 model in identifying SU posts. For this, we randomly sampled 3150 positive predictions from our customized model and queried GPT-3. Essentially, we designed a GPT-3 prompt, “Is this tweet <a real tweet post> related to substance use: Yes or No?” and queried for all sampled posts. Then, the predictions made by our model and GPT-3 were evaluated by our human experts.

### Ethical Considerations

To ensure the privacy and confidentiality of individuals whose data were analyzed, all study data underwent a rigorous deidentification process before analysis. The data for this study were sourced from publicly available platforms [63] containing no personally identifiable information. In addition, all the sample posts were preprocessed, removing user IDs, emails, URLs, numbers, stop words, and lemmatizing, making the resulting tokens impossible to identify users’ information. Thus, there was no personal information, including author names or any other private information, in the dataset. By addressing these ethical considerations, we conducted insightful research on SU patterns using social media content. In addition to this, our research was supported by the Substance Abuse and Mental Health Services Administration Strategic Prevention Framework-19 (grant 6H79SP081502), which was approved by the institutional review board at Kent State University (IRB20-182).

## Results

### Overview

Our primary objective was to comprehensively analyze the trends and patterns of SU over 3 years. To identify SU posts, we developed a self-trained deep learning model that achieved a precision rate of approximately 80%. This model was then used to detect SU tweets. The yearly breakdown of identified posts revealed 2,854,023 posts in 2019; 3,519,032 in 2020; and 2,567,970 in 2021. The identified data underwent various quantitative and qualitative analyses, such as trend analysis, k-means cluster analysis, topic and theme analysis, and factor analysis. To enhance the robustness of our findings, we validated our results by comparing them with those obtained from a GPT-3 model [79]. In the final section of our results, we present the outcomes of our integrated real-time application, showcasing the practical implications of our analyses.

### Trend Analysis (Question 1: How Did the Discourse on SU Evolve on Twitter From 2019 to 2021, and What Variations Existed in the Distribution of Different Substances During This Time?)

**Figure 5:**
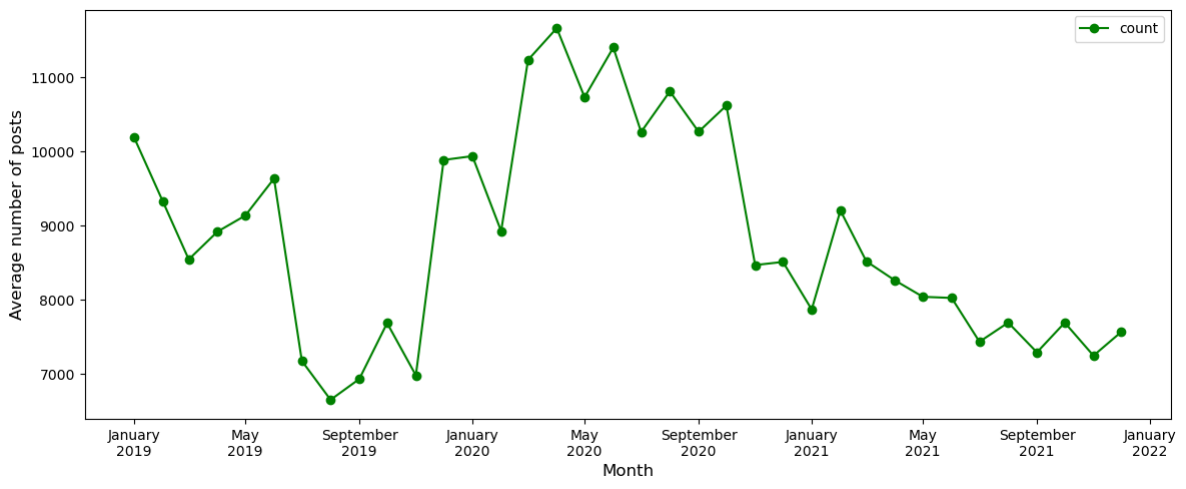
Substance use distribution from 2019 to 2021.

We began our research by conducting a time series analysis to understand the SU trend in the following 3 periods: the prepandemic period, the pandemic period, and the postpandemic period. We aggregated identified SU posts monthly to plot in the chart. Figure 5 shows the average number of SU posts for the entire study period. The proportional representation of the same chart can be found in Figure S1 in Multimedia Appendix 1. While the trend in average number seems substantially high in 2020 in comparison to pre- and postpandemic periods (Figure 5), the proportion of posts for the same data is observed to decline from 2019 to 2021.

**Figure 6:**
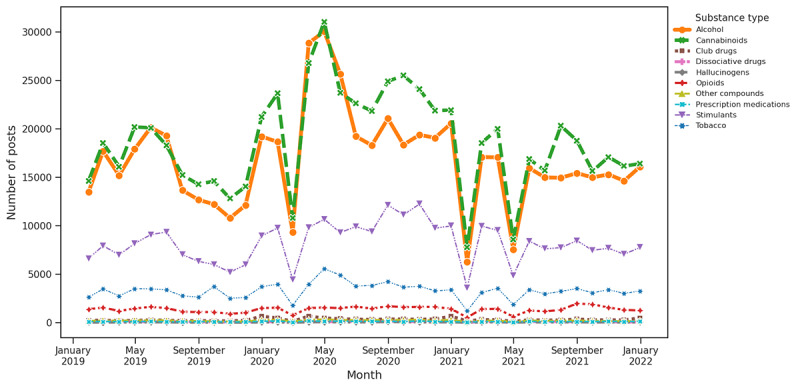
Substance type distribution from 2019 to 2021.

In addition, we also plotted a time series for 10 different substance categories to learn the trend of substances by categories. Thus, at first, we categorized each of the posts by applying a keyword-based method by referring to standard keywords from the NIDA [65], as outlined in Table S1 in Multimedia Appendix 1. For instance, we marked the post as alcohol if it contained any keywords associated with it and so forth. After the classification, we plotted the distribution for each substance type to visually understand the trend of each substance type in the study period, as shown in Figure 6.

### LDA Topic Analysis (Question 2: Following the Announcement of the Pandemic, What Were the Primary Substance Types That Garnered Significant Discussion, and What Were the Themes of These Dialogues?)

#### Drug Distribution 7 Days Before and After the Pandemic Declaration Day

Following the announcement of the pandemic on March 15, 2020, by Donald Trump, our result evidenced a significant 21% surge in the mentions of SU in tweets in just 3 days. Thus, understanding the change in pattern during that period was essential. We selected data from 7 days before and after March 15 to learn the impact of the pandemic declaration date on the trends. Thus, we aggregated the post count by each substance type 7 days before and after March 15, as shown in Table 2. The time series plot for the same period is also provided in Figure S3 in Multimedia Appendix 1.

**Table 2:** Substance type distribution 7 days before and after the pandemic declaration day.

Period and substance type		Proportion of posts, n (%)
**Seven days before March 15, 2020 (n=54,671)**		
	Tobacco	2165 (3.96)
	Alcohol	15,620 (28.57)
	Cannabinoids	23,837 (43.6)
	Opioids	1345 (2.46)
	Stimulants	10,241 (18.74)
	Club drugs	1000 (1.83)
	Dissociative drugs	98 (0.18)
	Hallucinogens	87 (0.16)
	Other compounds	470 (0.86)
	Prescription medications	98 (0.18)
**Seven days after March 15, 2020 (n=56,773)**		
	Tobacco	1936 (3.41)
	Alcohol	19,661 (34.63)
	Cannabinoids	21,341 (37.59)
	Opioids	835 (1.47)
	Stimulants	7914 (13.94)
	Club drugs	613 (1.08)
	Dissociative drugs	131 (0.23)
	Hallucinogens	57 (0.1)
	Other compounds	199 (0.35)
	Prescription medications	2606 (4.59)

#### LDA Topic Analysis

Furthermore, to comprehend the nuances of keywords and topics discussed following the declaration of the pandemic, we conducted an LDA topic analysis on these periods, 7 days before and after the official declaration. As shown in Tables 3 and 4, we highlighted the 10 main topics along with the distribution of the posts across each topic. Also, each topic consisted of the topmost terms that were extracted, excluding stop words.

**Table 3:** Top 10 terms of 10 latent Dirichlet allocation topics (7 days before the pandemic declaration day).

Topic	Top 10 terms	Distribution (n=54,671), n (%)
0	wine, buy, smoking, glass, everyone, water, red, drink, taste, beer	2733 (5)
1	alcohol, virus, corona, people, cigarette, leave, amp, covid, roll, hit	2733 (5)
2	beer, know, thing, fuck, try, man, cancel, cold, drink, problem	2733 (5)
3	drunk, bar, get drunk, blunt, pain, hold, kill, tonight, sick, coronavirus	2733 (5)
4	liquor, high, store, week, without, bitch, right, keep, always, low	2733 (5)
5	use, coke, would, call, drink, put, lmao, shot, really, enjoy, alcoholic	30,069 (55)
6	smoke, drink, drinking, sleep, drug, coffee, bro, work, fire, outside	2733 (5)
7	crack, night, love, last, stay, damn, smoke, cocaine, end, next	2733 (5)
8	weed, good, need, come, tequila, shit, smoke, drink, day, first	2733 (5)
9	nose, alcohol, bottle, year, please, beer, well, drink, hope, time	2733 (5)

**Table 4:** Top 10 terms of 10 latent Dirichlet allocation topics (7 days after the pandemic declaration day).

Topic	Top 10 terms	Distribution (n=56,773), n (%)
0	come, smoking, back, man, alcoholic, street, eye, way, chinese, sell	1947 (3.43)
1	drink, drinking, beer, bottle, sleep, tequila, good, drive, tonight, nose	1947 (3.43)
2	liquor, stop, alcohol, store, order, close, bar, help, essential, turn	1947 (3.43)
3	drink, cigarette, smoke, eat, tell, weed, food, hold, even, talk	1947 (3.43)
4	fuck, virus, coke, year, high, shot, corona, covid, people, kill	1947 (3.43)
5	high, last, blunt, night, please, lit, amp, loudlycryingface, thought, die	1947 (3.43)
6	smoke, shit, start, open, feel, find, lmfao, woozyface, asf, miss, fire	1947 (3.43)
7	quarantine, pain, crack, really, use, cocaine, damn, bitch, drug, liquid	39,196 (69.04)
8	wine, would, weed, see, glass, need, someone, lmao, drunk	1947 (3.43)
9	drunk, get, love, friend, house, home, free, eat, wine, stay	1947 (3.43)

### Theme Trend Analysis (Question 3: How Did the Prevalence of the Studied Theme Influence Various Types of Substances Used During the Studied Period?)

**Figure 7:**
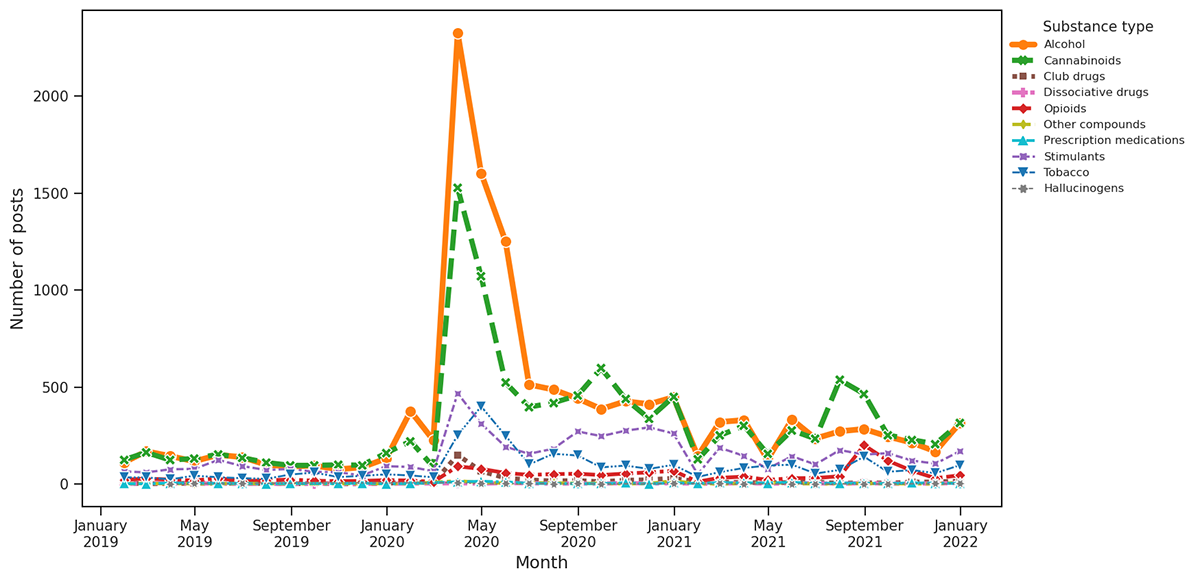
Substance distribution based on keywords associated with COVID-19.

**Figure 8:**
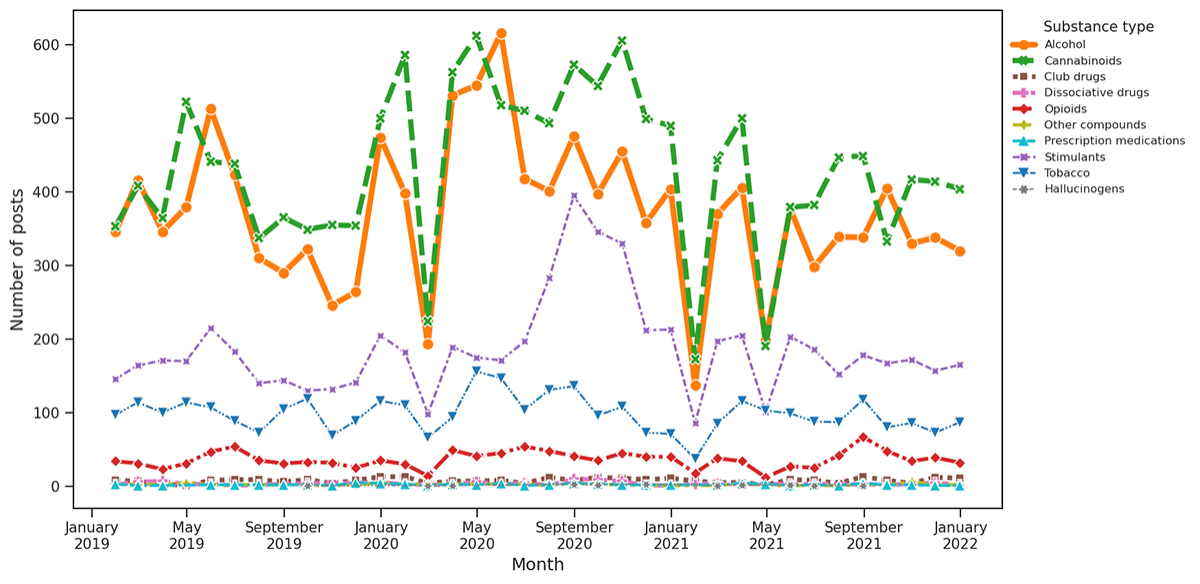
Substance distribution based on keywords associated with economic stress.

**Figure 9:**
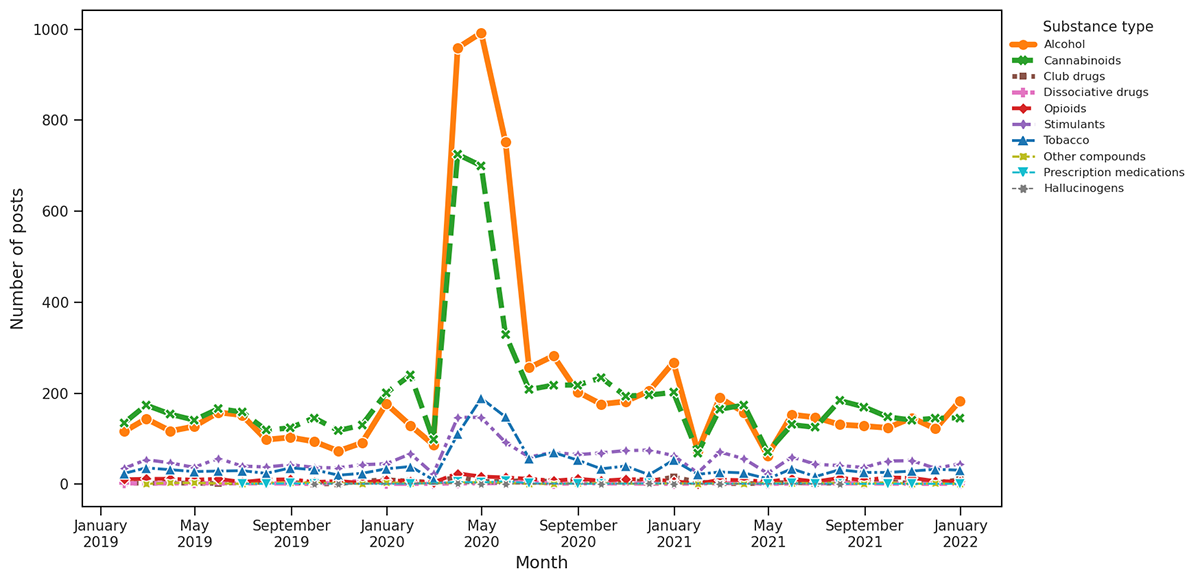
Substance distribution based on keywords associated with social stress.

**Figure 10:**
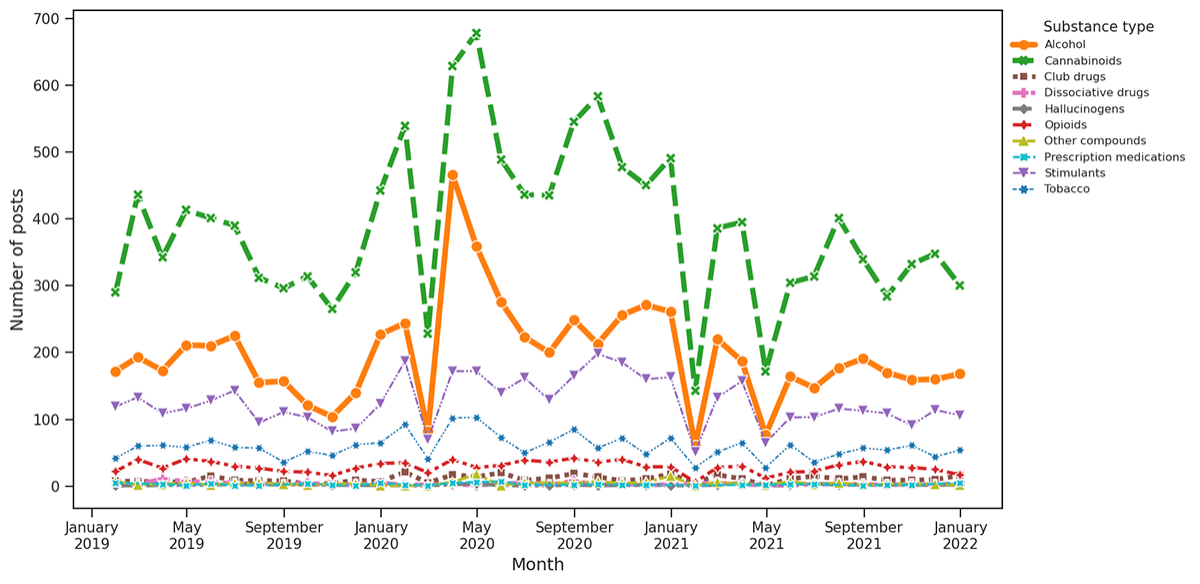
Substance distribution based on keywords associated with mental health.

**Figure 11:**
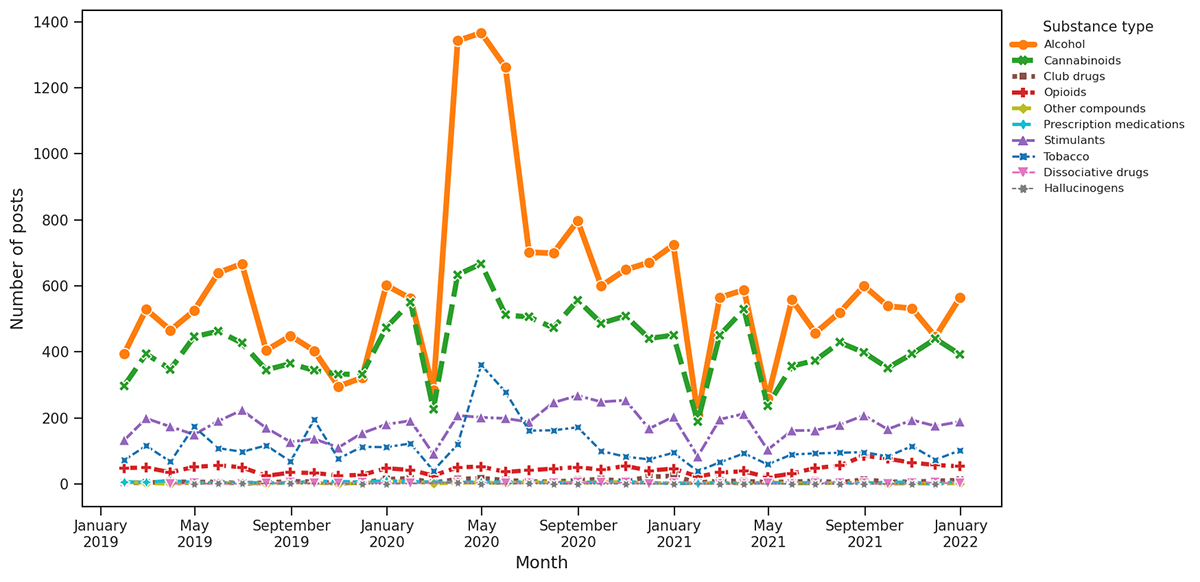
Substance distribution based on keywords associated with supply disruption.

**Figure 12:**
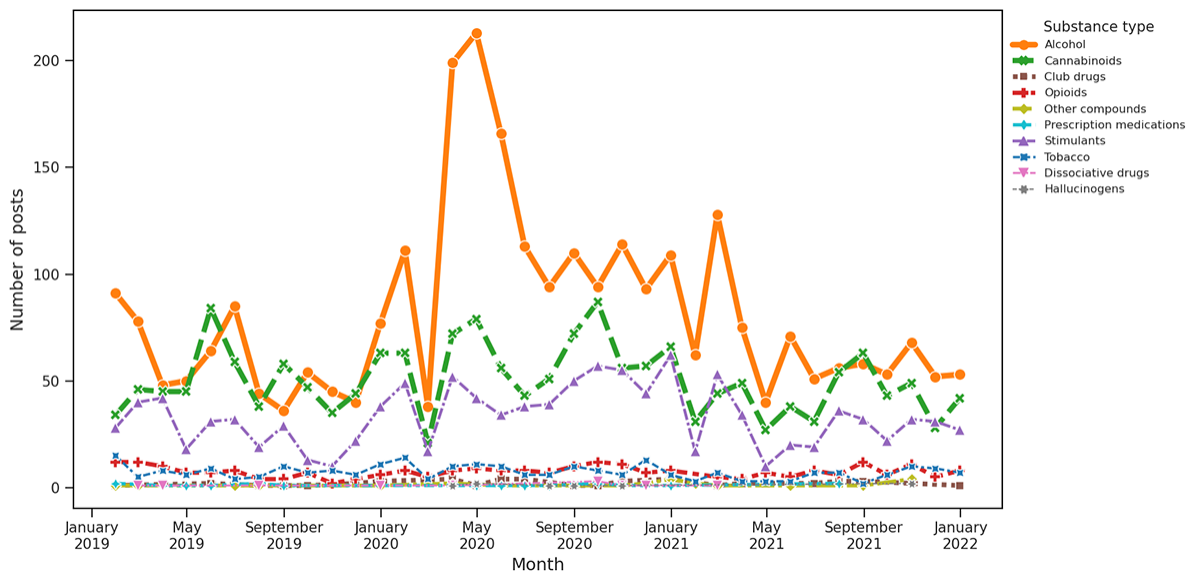
Substance distribution based on keywords associated with medical disruption.

The theme in any subjective study is either a topic or a related subject name that best describes the group of the data. In our context, we wanted to identify such themes in the SU posts so that we could analyze the pattern and further investigate a correlation with different substances. Thus, we derived 6 major themes, namely COVID-19, economic, social, mental health, supply disruption, and medical disruption, as discussed in the Baseline Themes section in the Methods section. Subsequently, we plotted a time series for each substance type for all themes, as depicted in Figures 7-12. Our research yielded valuable insights through a trend analysis focused on the impact of prevalent themes on SU.

### Thematic Analysis (Question 4: How Did the Identified Themes Correlate With the Substance Types?)

We performed heat map analysis and factor analysis to explore the correlation between identified themes.

#### Heat Map Analysis

**Figure 13:**
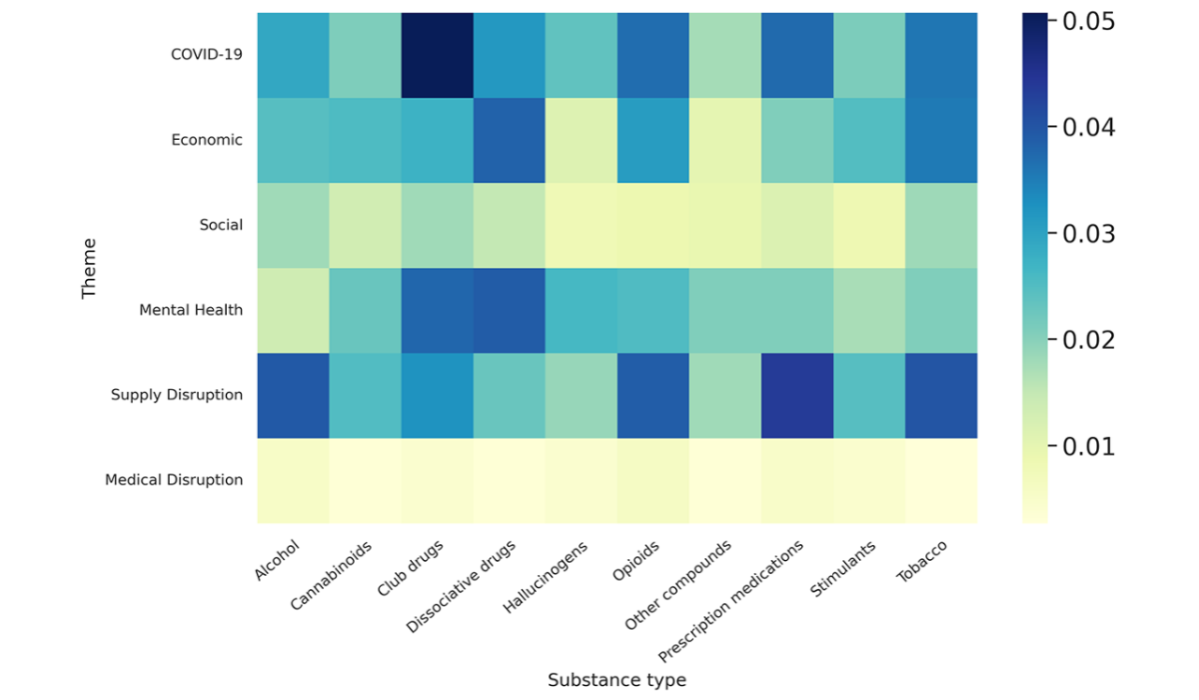
Heat map between themes and substance types.

In our study, we further used a heat map to visually analyze the relationships between identified themes (COVID-19, economic, social, mental health, supply disruption, and medical disruption) and substance types (alcohol, cannabinoids, club drugs, dissociative drugs, hallucinogens, opioids, other compounds, prescription medications, stimulants, and tobacco). The correlation plot is shown in Figure 13, where themes are represented on the y-axis and substances are represented on the x-axis. Each cell within the grid corresponds to a unique pairing of theme and substance type, with the color intensity indicating the strength of the association between them, and the color scale positioned along the right side of the vertical axis represents the intensity of association between these variables. Here, deeper shades of blue signify stronger associations, while lighter shades, reminiscent of lime, indicate weaker associations.

#### Factor Analysis

**Figure 14:**
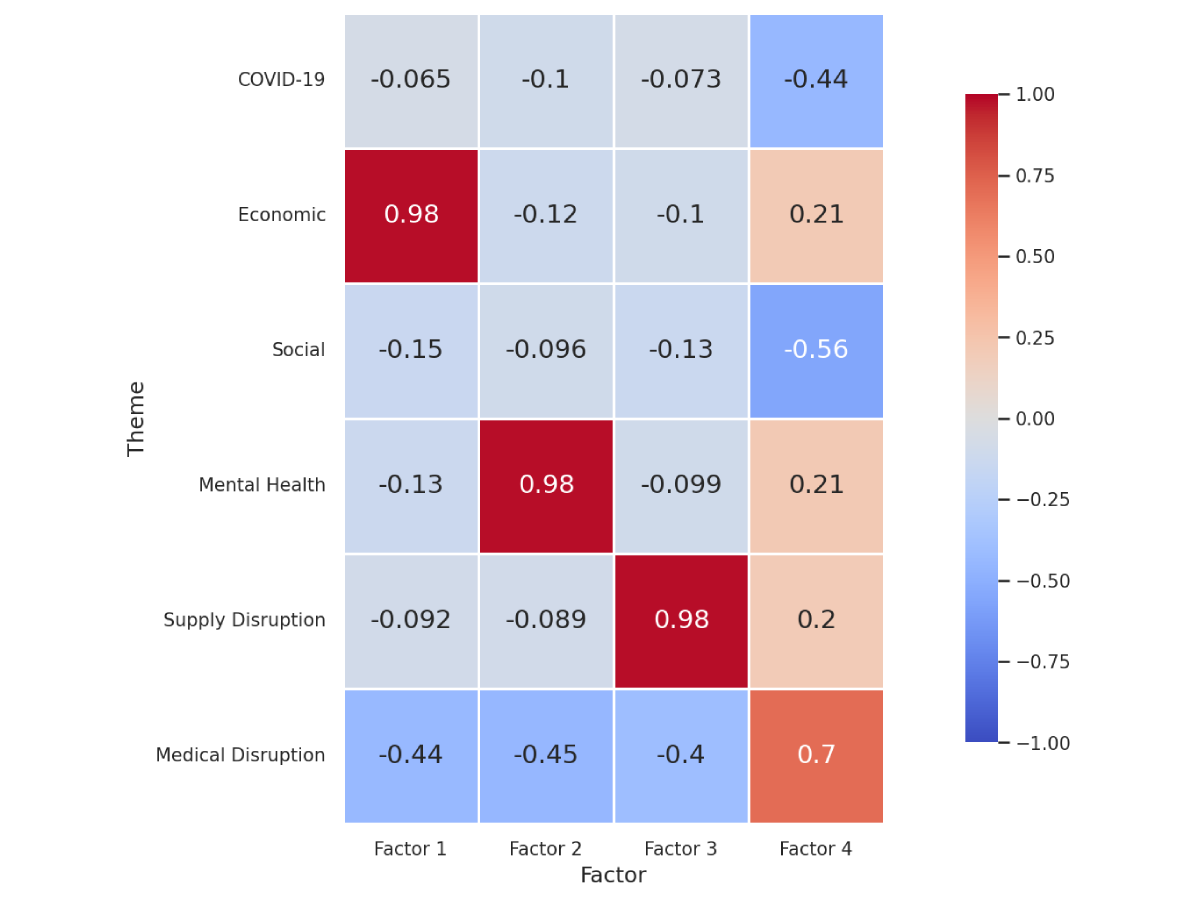
Factor loading heat map.

We performed factor analysis to examine the variability among the selected themes, aiming to distill these into a smaller set of unobserved, underlying variables known as factors. We determined the optimal number of factors to be 4 based on the Kaiser criterion, a decision further substantiated by the scree plot analysis, which revealed a distinct elbow point (Figure S5 in Multimedia Appendix 1). This analysis was facilitated by the *factor_analyzer* package within the Python application programming interface [45], which calculated the eigenvalues for each factor corresponding to the identified themes. The resultant factor loading heat map is shown in Figure 14. This heat map illustrates the relationships between factors and themes; negative values signify an inverse relationship, while positive values denote a direct relationship. The intensity of the relationship is indicated by values approaching 1 or –1 for strong relationships and values near 0 for weak ones. The heat map uses a color gradient where red shades indicate positive associations and blue shades indicate negative associations, providing a clear visual representation of these relationships.

### k-Means Clustering Analysis (Question 5: What Primary Discussion Topics Arise From k-Means Analysis, Specifically During the Pandemic Year?)

**Figure 15:**
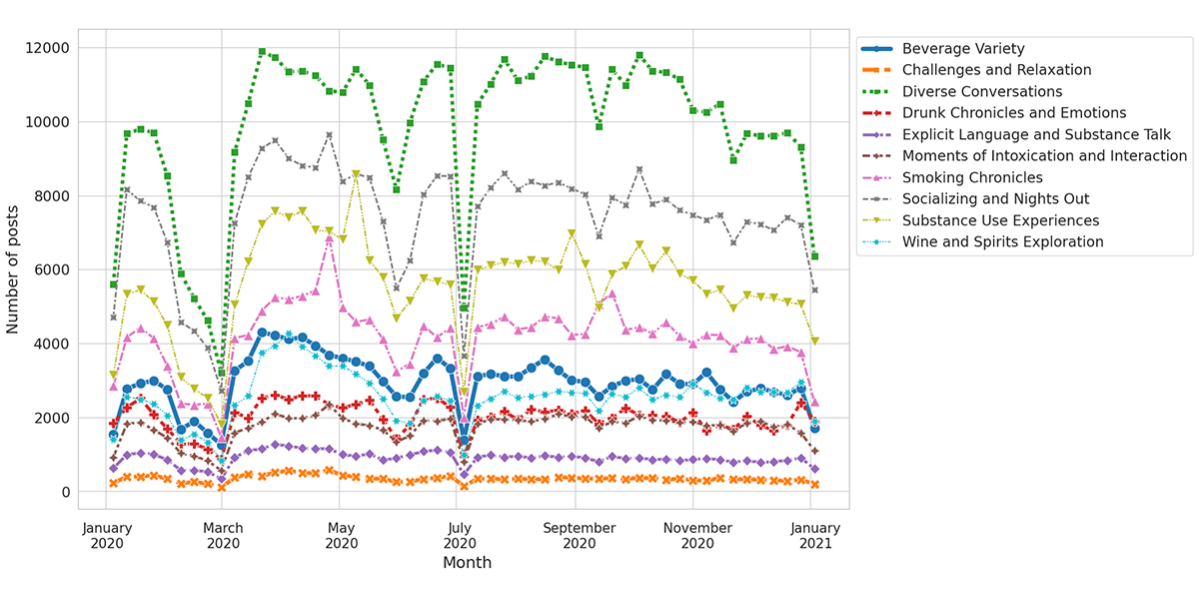
K-means cluster analysis of substance use posts in 2020.

In addition, we also performed k-means clustering on SU posts from 2020 to identify the relevant groups or clusters by leveraging the similar distance algorithm inbuilt in the k-means clustering method [72]. Essentially, we started by applying the elbow method to determine an optimal cluster size, which turned out to be 19 for our data. The elbow diagram is depicted in Figure S4 in Multimedia Appendix 1. Furthermore, we applied k-means clustering to generate 19 clusters. However, due to redundant cluster keywords, we merged the relevant clusters, resulting in 10 main clusters, as shown in Figure 15. The cluster keywords and their respective details are presented in Table S8 and Figure S4 in Multimedia Appendix 1, respectively. Initially, information from each cluster was gathered and categorized into interaction, discussion, feelings, and perceptions. In addition, correlated clusters were amalgamated, such as cluster 0 and 13 labeled as raw conversations—explicit language and substance talk; cluster 1 and 6 as smoking chronicles—weed and cigarettes; cluster 2 and 12 as wine and spirits exploration; cluster 4 and 9 as social highs—moments of intoxication and interaction; cluster 5 and 16 as socializing and nights out—drinks and smokes; cluster 7 and 8 as beverage variety—beers, alcohol, and voting; cluster 10 and 14 as SU experiences—smoking, drugs, and liquor; and cluster 15 and 18 as challenges and relaxation—tiredness, and blunts. Cluster 3, encompassing diverse experiences and activities, was named diverse conversation.

### Comparison With GPT-3 (Question 6: To What Degree Does the Classifier’s Effectiveness in Pinpointing SU-Related Tweets During the Pandemic Align With or Differ From GPT-3?)

We also compared our results with the GPT-3 model by asking the model to classify tweets using GPT application programming interface [79]. We used a prompt, “Is this tweet ‘<a real tweet post>’ related to substance use: Yes or No?” For this, we randomly sampled 3150 predicted positive results from our customized model and cross verified with the human (experts) and a machine (GPT-3). The human-verified 95.23% (3000/3150) of these predicted positive tweets were accurate, while GPT-3 only verified 53.73% (1693/3150) of the tweets as accurate. From this result, we concluded that generic powerful models such as GPT-3 do not necessarily generate true results when identifying hidden contexts in domain-specific data. This necessitates the need for domain-specific models for accurate results.

### Real-Time Application (Question 7: How Has the Overall System Contributed to the Real-Time Tracking of SU, as Evidenced by Research?)

**Figure 16:**
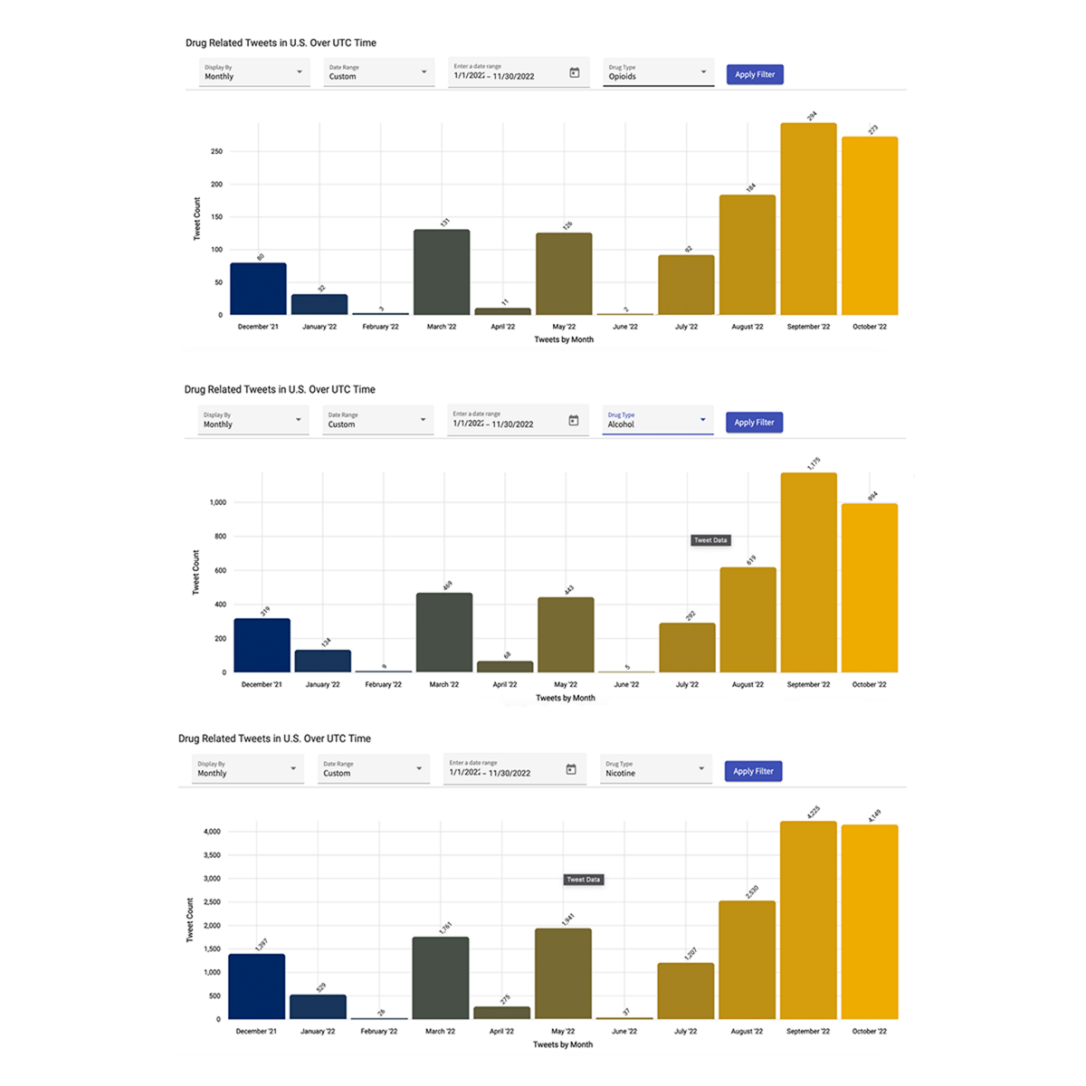
Snapshots of integrated real-time application. UTC: Universal Time Coordinated.

We further deployed our model to provide a real-time service in an application, Northeast Ohio Tri-County Prevention Infrastructure [80], specifically within the social media section designed for Ohio state. Primarily, the aim of the application was to serve as a monitoring and prevention dashboard for the state from static data. However, the real-time nature of social media data gave the application true power to monitor patterns of SU across areas of interest. Figure 16 provides snapshots of the application, illustrating how the stakeholders can dynamically monitor the SU segmented by time and substance type.

## Discussion

We used our custom deep learning model and several statistical methods to perform this analysis to get insights into the trends and impacts of COVID-19 related to SU. The subsequent sections elaborate on the results in detail.

### Trend Analysis

#### Time to Event Analysis and Substance Distribution for Question 1

The analysis of time to event reveals a significant increase in SU tweets in 2020, surpassing the counts for 2019 and 2021 by 17.6% and 22.35%, respectively (Figure 5). Notably, March 2020, April 2020, and June 2020 emerged as the focal months for SU discussions, with frequencies 16.55%, 21.18%, and 18.19% higher than other months in 2020. The elevated trend persisted until October 2020, likely coinciding with the availability of vaccines, highlighting a limitation in our study.

The examination of substance discussions over a 3-year time frame revealed a consistent focus on alcohol and cannabinoids, emerging as the predominant topics throughout the study. An intriguing observation during the pandemic period was the discernible surge in discussions surrounding alcohol, cannabinoids, and stimulant drugs, distinguishing them with an upward trend. In contrast, other substances did not exhibit substantial shifts in discourse.

It is crucial to exercise caution when interpreting the data for February 2020, January 2021, and April 2021, as the graph may be skewed due to limited available tweets in the Twitter source during those specific months. Despite this limitation, the broader insights gleaned from the study underscore the enduring prominence of alcohol and cannabinoids in public discourse. The pandemic period, marked by unprecedented global challenges, evidently influenced a notable increase in discussions surrounding these substances and stimulant drugs, indicative of evolving societal dynamics and coping mechanisms. These findings prompt further exploration into the nuanced factors shaping substance-related discussions, offering valuable insights for public health considerations and policy implications.

#### Topic Analysis and Substance Distribution for Question 2

In order to observe the impact of the declaration of the global COVID-19 pandemic declaration day on March 15, we analyzed the posts by each substance type 7 days before and after March 15. The aggregated posts in these 2 weeks had distinct changes in each substance type. Notably, discourse in only 2 substance types, alcohol and prescription medication, were observed significantly increasing, while discourse in all other substance types were observed slightly declining. The trend can also be visualized in Figure S3 in Multimedia Appendix 1. The increased trend of alcohol discussion was likely due to the effect of COVID-19, particularly due to closed schools, social isolation, boredom, and various types of mental stress and anxiety, which is also supported by some studies [15,35,36]. A study by Farhoudian et al [15] that conducted a survey in May highlighted the increment in alcohol, prescription medication, and cannabinoids. However, in our study, cannabinoids showed a slight decrement in a 7-day period while it remained significantly discussed during the entire study period.

Moreover, our topic analysis for the same period indicated a shift in substance-related discourse. In general, during the prepandemic period, references to substances were casual in almost all the 10 topics, as depicted in Table 3. Although topic 5 had the highest proportion of keywords (30,069/54,671, 55%), the terms referred to casual keywords, insignificant to any particular substances or behavior. However, topics in the postpandemic period included keywords that concerned quarantine and SU as seen in topics 4 (1947/56,773, 3.43%) and 7 (39,196/56,773, 69.04%) in Table 4. The mention of keywords (such as *nose*, *coronavirus*, and *covid*) during the first period suggested that COVID-19 has been interlinked with few substance discussion; however, there were no negative words indicating stress or bad impact on mental health. By contrast, the topics in the second period included negative keywords (such as *pain*, *die*, *stress*, and *fuck*) along with SU keywords. This shift suggests a nuanced decline in mental health after the pandemic declaration day. Likewise, topics 1, 2, 8, and 9 in the second week contained more alcohol- and liquor-related keywords (such as *drink*, *beer*, *bottle*, *liquor*, *store*, and *drunk*), suggesting use of alcohol as the main substance during this period. Nevertheless, there were no major terms in the topic analysis that could support prescription medication use in the second week.

In conclusion, our detailed analysis on 7 days before and after the pandemic declaration day highlights the immediate impact on the use of substances, particularly alcohol and prescription medication.

#### Theme Trend Analysis and Substance Distribution for Question 3

As per our keyword-based theme analysis, COVID-19 had a notably significant impact on the discussion of SU. The early pandemic period showed a significant rise in alcohol and cannabinoids associated with 2 main themes as follows: COVID-19 and social isolation. This surge was most evident at the onset of the pandemic in early 2020, likely reflecting a response to the stress, uncertainty, and lifestyle changes imposed by the health crisis. The data indicated that these increases were particularly influenced by COVID-19–related factors, with social and economic aspects also playing a role. In contrast, factors related to supply and medical disruptions did not drastically affect use patterns. This concentrated spike in alcohol and cannabinoid use during challenging periods highlights the broader impact of the pandemic on SU behaviors.

#### k-Means Clustering Analysis for Question 5

From the k-means clustering, we identified 10 main clusters as an indication of what was discussed in the pandemic year as follows: beverage variety, challenges and relaxation, drunk chronicles and emotions, explicit language and substance talk, moments of intoxication and interaction, smoking chronicles, socializing and nights out, SU experiences, wine and spirits exploration, and diverse conversations. The diverse conversation cluster includes all the remaining tweets that do not belong to particular clusters. Hence, the number of posts in it has the highest counts. Excluding this cluster, SU-associated posts were mostly seen in socializing and nights out, followed by SU experiences and smoking chronicles as the 3 main top discussions.

#### Thematic Analysis for Question 4

##### Heat Map Analysis

The heat map analysis provided insightful revelations regarding the factors influencing SU discourse, highlighting COVID-19, economic stress, mental health concerns, and alterations in drug supply as the principal elements. Specifically, there is a stronger correlation between the “COVID-19” theme and cannabinoid use, possibly signifying an increase in this substance’s consumption as a direct response to the pandemic’s stressors. The “economic” theme shows a somewhat lower yet noticeable correlation with alcohol, which might reflect economic uncertainty’s impact on alcohol consumption. The “social” theme has a less pronounced correlation across all substance types, implying that social factors had a milder influence on SU during this period. “mental health” has a moderate correlation with both cannabinoids and alcohol, highlighting these as coping mechanisms during mentally challenging times. “supply disruption” shows a varied correlation but is not significantly linked with any substance, suggesting that supply issues did not drastically alter consumption patterns. Finally, “medical disruption” seems to have the least correlation with SU, suggesting that medical service disruptions during the pandemic had minimal influence on the consumption of these substances. Overall, the heat map indicates that COVID-19–related factors had the most significant correlation with changes in SU, with economic and mental health factors also being relevant but to a lesser extent.

##### Factor Analysis

The factor analysis gave insights into a combination of themes that had an impact on SU. Factor 1 indicated that mental health was the leading factor. Factor 2 was strongly and positively associated with the economic theme, suggesting that this factor could represent financial stress or economic consequences of the pandemic. The social theme had a moderate negative loading on factor 2, implying that social aspects may decrease in relevance as economic concerns increase or vice versa. Factor 3 showed a very strong negative loading with the medical disruption theme, indicating that this factor was significantly influenced by disruptions in medical services. This could represent the strain on health care systems and the impact of health care access on the population. Mental health and supply disruption themes had a strong positive loading on factor 4, implying that this factor may represent the psychological impact of the pandemic and its influence on drug supply chains.

In summary, the factor analysis suggested that economic and mental health themes were major dimensions of the pandemic’s impact, with medical disruptions also playing a significant but negatively associated role.

#### Comparison With GPT-3 for Question 6

Our comparative analysis with the GPT-3 model yielded valuable insights into the effectiveness of powerful generic models in identifying hidden contexts in domain-specific data, particularly related to drug use in tweets. The experiment involved using a prompt to classify tweets as either related or unrelated to SU. The results demonstrated a substantial discrepancy in accuracy between human verification and GPT-3. When comparing the randomly sampled predicted positive tweets, human experts confirmed the accuracy of 95.23%, whereas GPT-3 verified only 53.73% of the tweets as accurate. This notable difference underscores the limitations of generic models such as GPT-3 in accurately discerning domain-specific nuances. Although we have not performed a detailed analysis to find out the reason behind this discrepancy, we anticipate the limitation of contextual awareness as a primary reason for this, as indicated in the studies by Ray [81] and Moradi et al [82]. For instance, Moradi et al [82] highlighted similar cases where GPT-3 underperformed in the biomedical corpora in comparison to domain-specific pretrained model BioBERT [82]. By contrast, generic pretrained models such as ours can provide rich contextual understanding as they are pretrained solely on social media data, making them powerful in understanding slang-like languages. Thus, the limitation in GPT-3 is well addressed by our custom model pretrained on domain-specific data.

#### Real-Time Integration for Question 7

The successful integration of our trained model into the practical application Tri County Prevention Infrastructure [80], particularly within the social media section tailored for Ohio, marks a significant achievement. This integration empowers real-time users by allowing them to visually explore the distribution of substance-use posts in both temporal and spatial dimensions. For instance, the users can explore and analyze the trend of any substance (eg, alcohol) in real time and take immediate actions to mitigate the use in the areas of interest. In addition, the applicability of our models’ integration is promising during crisis periods such as the COVID-19 pandemic, when physical intervention is unfeasible.

### Limitations

Our study has several limitations. Initially, data inconsistencies in certain months were due to incomplete datasets from the sources [63]. Moreover, our analysis was confined to English-language posts, potentially excluding non-English speaking users and thus not reflecting the full spectrum of users during the study period. The initial annotated data used for the training model were collected from a specific time frame (January 2020 through April 2020). The selection of data from this particular time frame could have introduced some bias in the SU identification process. In addition, the consideration of precision as our primary evaluation metric during iterative fine-tuning steps could have missed real SU posts, limiting to the small spectrum of patterns learned by the model and leading to overfitting. Also, the overall accuracy of the classifier reached 80%, which could have led to non-SU posts being identified as SU posts and vice versa. Consequently, this could actually deviate the count of SU posts identified in our study, thus deviating from trend studies. Although the choice of classifier, RoBERTa, seems to have performed better, the identification of SU tweet posts for multiple sequences could have been misclassified. Likewise, the limited labeled dataset during fine-tuning could have underfitted the performance in the initial rounds. While we used HITL [61] in our iterative fine-tuning approach to enrich the annotated data, the human reviewers involved in the process were only tasked with reviewing model predictions without providing feedback. This lack of active human feedback may have limited the model’s capacity for improvement as corrections to errors and mispredictions or rewarding accurate predictions could have enhanced its performance further. In the future, incorporating a full HITL at different stages of model development could significantly improve accuracy and model refinement. Finally, the scope of keywords used in processing tweet data may have been too narrow, possibly leading to an overrepresentation of certain themes and factors in our results.

### Future Work

This study only considered text data for the identification of SU. Future research could use multimedia, such as images and videos, to enhance the accuracy of the identification of SU. Furthermore, our iterative fine-tuning approach could be enhanced through active learning [62], where the most critical samples are selected for annotation in each iteration, optimizing model performance. Another potential improvement involves incorporating full HITL feedback [61], allowing human reviewers not only to review but also to correct errors or reward accurate predictions. This approach could significantly refine model accuracy. In addition, a user-level analysis could be conducted to investigate factors influencing the intention and purpose behind substance misuse. In addition to this, demographic factors such as age, gender, race, emotion, socioeconomic status, personality trait, and mental and physical health status could be considered for investigation to understand the most impacted cohort during the pandemic. By understanding these cohorts and factors, we can develop strategies and interventions to prevent and control SU during global crises.

### Conclusions

In this study, we conducted an extensive infodemiology analysis of Twitter posts from 2019 to 2021, focusing on SU patterns during the COVID-19 pandemic. Using a deep learning model (RoBERTa) alongside techniques with human involvement in iterative fine-tuning, our classifier achieved an optimal accuracy of 80%, even with limited resources. This performance is notable as even a powerful state-of-the-art model such as GPT-3 struggled with domain-specific data such as SU.

In summary, the results from our study showed the key patterns in SU trends during both the pandemic and overall study periods. The analysis of the pandemic period has shown that COVID-19 had a huge impact on the influx of SU. As indicated by trend analysis, the numbers were higher during the peak pandemic period, mainly between March and October 2020. Furthermore, the theme analysis showed a higher association of SU posts with COVID-19 and social themes in comparison to other themes during the pandemic period. In addition to this, the immediate declaration of the pandemic introduced stress and anxiety in public, as evidenced by our LDA topic analysis, causing a significant rise in SU (21% in just 3 days), primarily in readily available substances such as alcohol and prescription medication. These findings suggest that the authorities should pay attention to key factors such as social isolation, stress, and anxiety, and focus on strengthening regulations around the sale of accessible substances such as alcohol, prescription medications, and cannabinoids (though not legal in all areas) to have control the SU during the global COVID-19 pandemic crises. By contrast, economic, mental health, and supply disruptions seem to be the major contributing factors for SU throughout the study period, as indicated by our factor analysis, with cannabinoids, alcohol, and stimulants as dominating substances. Thus, public health agencies should focus on controlling the economic and mental health of global citizens as key actions, alongside surveilling drug supplies, in order to control global SU.

In summary, our study demonstrates the applicability of social media data used along with a deep learning model to analyze trends in global issues such as SU. The findings and methodology from this study can help public health sectors develop real-time strategies and prevent SU during future crises.

## Appendix

Multimedia Appendix 1 Additional dataset details, classified tweet samples, and analysis figures to support this paper’s investigation.

## Abbreviations

BERT: bidirectional encoder representations from transformers
HITL: human-in-the-loop
LDA: latent Dirichlet allocation
MLM: masked language model
NIDA: national institute on drug abuse
NLTK: Nature Language ToolKit
NSP: next sentence prediction
RoBERTa: robustly optimized bidirectional encoder representations from transformers pretraining approach
SU: substance use
SUD: substance use disorder

## Data Availability

The data and code supporting this study are publicly available on GitHub [83].
