# Supplementary material for: Large-Scale Deep Learning–Enabled Infodemiological Analysis of Substance Use Patterns on Social Media: Insights From the COVID-19 Pandemic
Source: JMIR Infodemiology. 2025 Apr 17;5:e59076. doi: 10.2196/59076 (PMC12046268; doi:10.2196/59076)
Supplement: Multimedia Appendix 1 [file infodemiology_v5i1e59076_app1.docx]

Deep Learning–Driven Insights into Substance Use During the COVID-19 Pandemic from Social Media Data

## **1 Data and Methods**

### **1.1 Substance types and their associated keywords**

The following table presents the associated keywords and slang words for each substance type used in the study.

Table S1. Substance types and their associated keywords

| **Substance Type** | **Keywords** |
| --- | --- |
| tobacco | nicotine, tobacco, cigarette, cigars, bidis, snuff |
| alcohol | liquor, beer, wine |
| cannabinoids | marijuana, blunt, dope, ganja, grass, herb, joint, bud, mary jane, pot, reefer, green, trees, smoke, sinsemilla, skunk, weed, hashish, boom, gangster, hash, hash oil, hemp |
| opioids | heroin, smack, horse, brown sugar, dope, H, junk, skag, skunk, white horse, China white, opium, laudanum, paregoric, big O, black stuff, block, gum, hop |
| stimulants | cocaine, hydrochloride, blow, bump, C, candy, Charlie, coke, crack, flake, rock, snow, toot, amphetamine, Biphetamine, Dexedrine, bennies, black beauties, crosses, hearts, LA turnaround, speed, truck drivers, uppers, methamphetamine, Desoxyn,meth, ice, crank, chalk, crystal, fire, glass, go fast, speed |
| club_drugs | mdma, ecstasy, adam, clarity, eve, lovers speed, peace, uppers, flunitrazepam, rohypnol, forget-me pill, mexican valium, r2, roach, roche, roffles, roofinol, rope, rophies, ghb, Gamma-hydroxybutyrate, G, Georgia home boy, grievous bodily harm, liquid ecstasy, soap, scoop, goop, liquid X |
| dissociative_drugs | ketamine, Ketalar SV, cat Valium, K, Special K, vitamin K, pcp and analogs, phencyclidine, angel dust, boat, hog, love boat, peace pill, salvia divinorum, salvia, shepherdess’s herb, maria pastora, magic mint, sally-d, dextromethorphan, dxm, robotripping, robo, triple |
| hallucinogens | Lsd, Lysergic acid diethylamide, acid, blotter, cubes, microdot, yellow sunshine, blue heaven, mescaline, Buttons, cactus, mesc, peyote, psilocybin, Magic mushrooms, purple passion, shrooms, little smoke |
| other_compounds | anabolic_steroids, anadrol, oxandrin, durabolin, depo-testosterone, equipoise, roids, juice, gym candy, pumpers, inhalants, Solvents, paint thinners, gasoline, glues, gasses, butane, propane, aerosol propellants, nitrous oxide, nitrites, isoamyl, isobutyl, cyclohexyl, laughing gas, poppers, snappers, whippets |
| prescription_medications | cns_depressants, stimulants, opioid pain relievers, oxyContin, oxycodone, vicodin, norco and lortab, hydrocodone, acetaminophen, Percocet, oxycodone, acetaminophen, Tramadol, Codeine, morphine, methadone, Demerol, meperidine, acetaminophen, tylenol, excedrin, vanquish, aspirin, Bayer, bufferin, ecotrin, excedrin, vanquish, diclofenac, voltaren Gel, ibuprofen, advil, motrin IB, naproxen, aleve |

### **1.2 Annotated sample tweets**

The following table shows sample tweets that are annotated by the experts as Substance Use or Non-Substance Use.

Table S2. Annotated tweet sample

| **Tweet** | **Substance Use** |
| --- | --- |
| @USER bring me a blunt pls | Yes |
| Been rolling this same blunt since opening my eyes at 6:50 | Yes |
| OH in the press room: "I was really confused about that -- where he was getting his heroin from." | Yes |
| Happy Birthday @USER !!!! We getting lit tonight! | Yes |
| How much coke she did no wonder age OD'd. Damn. | Yes |
| I do have Brown as my WR/RB flex, so, yayy?? #... | No |
| april was annoying but tolerable BUT THEN SHE GOT DEREK SHOT OHMYGOD FUCK APRIL TOO | No |
| im addicted to my manz😋😍idc, idc. | No |

### **1.3 RoBERTa Configurations**

The settings and hyperparameters used in the design and training RoBERTa model are provided below in Table S3 and S4.

Table S3. Model settings

| **Variable** | **Value** |
| --- | --- |
| tokenizer | ByteLevelBPETokenizer |
| vocab_size | 8192 |
| min_frequency | 2 |
| special_tokens | ["<s>", "<pad>", "</s>", "<unk>", "<mask>"] |
| max_position_embeddings | 514 |
| num_attention_heads | 12 |
| num_hidden_layers | 6 |
| hidden_size | 768 |

Table S4. Training hyperparameters

| **Variable** | **Value** |
| --- | --- |
| batch_size | 16 |
| epoch | 32 |
| learning_rate | 1e-4 |
| weight_decay | 0.01 |
| seed | 42 |
| max_len | 128 |

### **1.4 Theme and their associated keywords**

Table S5. Six major themes and corresponding keywords

| **Theme** | **Keywords** |
| --- | --- |
| covid | covid, corona, coronavirus, outbreak, spread, viral, virus, flu, infect, pcr test, national emergency, wuhan, CDC, ncov, cov2, SARS, mask, sanitizer, toll, new disease, news, positive, test, cases, dangerous year, fake year, fda, flatten, quarantine, lockdown, pandemic, epidemic, ventilator, symptomatic, incubation, transmission, immune, vaccine, hard to breath, shortness of breath, start nose, variant |
| economic | job, job loss, jobless, no job, lost job, income, salary, paycheck, money, bills, bill raise, check, saving, wage, fulltime, unemployed, economy, layoff, recession, stimulus, evict, laid, bankrupt, owe, afford, eviction, credit, debt, loan, financial crisis, inflation, poor, poverty, normalize risk, cost, homeless, rent, mortgage, no food, no shelter, wallet, hungry street, fda |
| social | lockdown, bully, loneliness, emptiness, lonely, distancing, insecurities, no one care, trap, feel ignore, ignored, bored, quarantine, alone, stay home, stayhome, stay safe, stayathome, single, solo, lockup, disconnect, silence, off limit, frayed, indoor |
| mental health | sad, angry, mad, tension, depress, tire, exhausted, fear, scared, stress, worry, disappear, selfharm, crisis, dizzi, no hunger, no sleep, no hope, hopeless, loneliness, no will, quit life, no motivation, no motif, no focus, no dedication, cannot focus, panic, panic attack, rapid heartbeat, heartbeat fast, suicidal thought, ending my life, no reason to live, feeling worthless, seeking a way out, feel like a burden, struggling to cope, lack of purpose, wanting the pain to stop, mental pain, mental anguish, feel pressure, feel trapped, desperation, broken inside, losing the will to live, dark thought, depression, anxiety, ptsd, bipolar, intrusive, trauma, burnout, grief, dyingfeel, sorrow |
| supply disruption | steal, deliver, refuse, small rate, sellout, sell bath, sell illegal, sell weed, cheap deal, box sale, sale, order, order pack, cut safe, strong, weak, legal high, dangerous, toxic, industry, contaminated, local, import, international, darknet, market, stock alert, batch, customs, seizure, permit, lack, shortage, crisis, sale, buy drug, supply |
| medical disruption | health crisis, emergency admission, admission drop, crisis, pharmacy, shut down, cut off, on hold, locked out, dead end, dry, mia, rehab, virtual, telehealth, teletherapy, telemedicine, appointment, close clinic, limited hours, waitlist, treatment full, medical line, freeze, overload, emergency, drought, no help line, customer service |

### **1.5 Sample Tweets with identified substance type**

The table presents the sample tweets identified as Substance Use posts by the RoBERTa model.

Table S6. Sample tweets with identified substance type

| **Tweet** | **Substance Type** |
| --- | --- |
| florida suspend alcohol consumption bar state announces record singleday increase nearly 9000 new coronaviru… | alcohol |
| taurus szn bitch let eat amp toast unheard wine pretty label bed | alcohol |
| walk away local brewer day insist wear mask order beer… | alcohol |
| buy father day card dad bald beer drink serial farter navigate minefield | alcohol |
| drinking alcohol cause terrible disease cancer make human sad new new disease ca… | alcohol |
| wine drunk merry thanksgiving | alcohol |
| jimin know run alcohol big mood | alcohol |
| pass alcohol drug test include clean record brake fail go hill completely company… | alcohol |
| agree great beer | alcohol |
| ny take action stop spread response rise covid number establishment state liquor license… | alcohol |
| patient lung cancer catch sneak behind ward smoke igbo be nigerian patient frustrate wearyface | cannabinoids |
| nah yeah never catch talk shit people life esp smoke cus… | cannabinoids |
| addict weed lol nice smoke wake up get shower also | cannabinoids |
| smoke signal fire | cannabinoids |
| doctor ask drink smoke say yes know go help you… | cannabinoids |
| smoke cousin mexico and | cannabinoids |
| eat jean jacket cover peanut butter smoke cig | cannabinoids |
| raise right mean drink party smoke raise right treat people man… | cannabinoids |
| “ aye come outside bouta smoke ” me | cannabinoids |
| still hard believe pop smoke dead | cannabinoids |
| never funny msnbc regular offend bloomberg describe oligarch man li… | club_drugs |
| mom amp sister leave middle night amp never come back father addict drug amp die | club_drugs |
| really need rooftop bar whisky bourbon cigar need destress properly | club_drugs |
| can not believe bitch run mayor ’ s main goal rile invigorate protester the… | club_drugs |
| let bitch kno fine every chance get nigga gas station get fucked | club_drugs |
| drinking get fuck up even drink | club_drugs |
| outside work smoking guy approach find job company reach full capacity terms… | club_drugs |
| bcos get hand out afl football public amp happy take it nearly ever… | club_drugs |
| even get coffee you | club_drugs |
| girl wana talk bout period pain ever roll ankle this | club_drugs |
| lorraine cox stalk murder devon last year illegal iraqi immigrant come boat france and… | dissociative_drugs |
| ketamine date rape drug | dissociative_drugs |
| would rather tube amp drink boat somewhere degree weather | dissociative_drugs |
| drop slight hint recreational ketamine use it | dissociative_drugs |
| get drunk boat today | dissociative_drugs |
| spce actually double triple quadruple unlike others high short lowe… | dissociative_drugs |
| party boat captain shirtless drunk | dissociative_drugs |
| bts ’ triple crown sb inkigayo crown dna crown fake love crown idol crown boy love crown crown dynamite crown life go crown butter new… | dissociative_drugs |
| rick ross easily besides boat cocaina lie sell claim bitch wrap legs around hi… | dissociative_drugs |
| happen smoky meet beatles syndicate boat row | dissociative_drugs |
| lemonlemonlemon eyeshadow buttercupcake acidberry midori liquid lipstick roc | hallucinogens |
| please call pick acid bucket google how keep blood liqui… | hallucinogens |
| spider get high build different kind web weed caffeine mescaline lsd | hallucinogens |
| use easily able drink bottle echo fall apple cactus jack night wake fresh | hallucinogens |
| nobody like drain terrible odor come it degrade grease fatty acid clog drain wi… | hallucinogens |
| paint town redtongue little prickly pear cactus fruit sell street marrakech good | hallucinogens |
| know harvest timescissors become brownish turn grey weed ener… | hallucinogens |
| roomate try figure acid weed give giggle | hallucinogens |
| instead give billion trump wall fix flint water crisis 55 mil feed homeless vet mealsday for… | hallucinogens |
| since daughter graduate homeschool two son still give great lit option thank you | hallucinogens |
| spiritual successor okami hope come plateforms apple arcade | opioids |
| fox try xmen origins hopefully good | opioids |
| camera smack sana ’ s face… cameraman look drunk would last day twice | opioids |
| bit top shout horse maybe horse rude her | opioids |
| oregon vote legalize possession heroin antifa seem notice vandalize starbucks https… | opioids |
| way get thru mercury retrograde wo one argument friendship end something revealed car pr… | opioids |
| take advantage people know drunk gross amp hope hell | opioids |
| get high horse know sort quickly | opioids |
| hope tell sh1te replace bar thiago problem be… | opioids |
| fool horse bar scene | opioids |
| cow milk gross drink gasoline | other_compounds |
| joe rogan gateway drug plastic bag full gasoline | other_compounds |
| another dirkjake doodle— why car cat movie teeth drink gasoline food source that… | other_compounds |
| trump say word “ ethanol ” too mean drink gasoline tank moron grow cut… | other_compounds |
| moment dusty living room gamer save family zombie droids drunken sorcerer… | other_compounds |
| jaebeom jinyoung handshake leave ahgases get drink 갓세븐 USER… | other_compounds |
| great job hillbilly mad pay drink gasoline | other_compounds |
| know right much cheap buy liter gasoline | other_compounds |
| goto yogurt drink indeed nourishment body act gasoline engine kindly ha… | other_compounds |
| man drinking gasoline | other_compounds |
| mexico really legalize cocaine | stimulants |
| idc cold outside still gonn drink ice coffee | stimulants |
| crack | stimulants |
| think time crack | stimulants |
| can not even blow nose without think | stimulants |
| even coke bore camp | stimulants |
| pablo escobars son reveals dad work sell cocaine medium silent | stimulants |
| jungkook get drunk jungkook think people nice respect other | stimulants |
| mama adunni put smile people face house make fit get chicken drink house rice dey | stimulants |
| back 2011 blonde patch hit street harder crack 80 ’ s | stimulants |
| yes cele look two share cigarette hidden lunch box wonder get the… | tobacco |
| hello alanna bledman solemnly swear give smoking day night see cigarette | tobacco |
| new ashtray cigarette | tobacco |
| never want trade place cigarette fuck bad | tobacco |
| new questionif really care adverse impact cigarettes health wh… | tobacco |
| pov listen cigarette sex think crush overwhelmingly sad | tobacco |
| like alex smith radiohead mac demarco modern baseball weezer neutral milk hotel cigarettes se… | tobacco |
| eric garner kill sell cigarette | tobacco |
| year year one item get tax heavily without fail cigarette so highly doubt the… | tobacco |
| new men admit role scheme money cigarette offer homeless people skid row in… | tobacco |

### **1.6 Sample Tweets with identified theme**

Table S7. Sample tweets with identified themes

| **Tweet** | **Theme** |
| --- | --- |
| corona virus flu virus miley cyrus idgaf bet close liquor store | covid |
| say year ago smoke kill guess believe thing covid | covid |
| wendy lanski survive covid take drug tout trump part treatment get ongoing rapi… | covid |
| corona virus get people horny depressed creative true alcoholicsi | covid |
| day quarantine wine become new favorite drink | covid |
| waste money time pandemic thousand unemployed tell eat drink inside resta… | economic |
| donald right money cdc claim new covid case people wear mask … | economic |
| snide alcoholsoaked subversive flew crosscountry ensure american people get financial relief from… | economic |
| sugar daddy use get drunk emotional tell like money uhh doiiiiiiiiii | economic |
| never forget job growth donald trump slow obamabiden—even covid | economic |
| tire asl smoke amp sleep get wearyface | mental health |
| home get drunk damn sad | mental health |
| bro rehab currently fight heroin addiction fight mental health and… | mental health |
| literally feel pain forget bout life drunk life | mental health |
| pree tire get put shroom | mental health |
| stay home long get imaginary kid catch one smoking | social |
| cancel quarantine drunk skeleton halloween shirthappy quaro halloween | social |
| quarantine routine pour drank headshot drink sit drank … | social |
| social distancing front wine shop | social |
| sweet leave alone though cuz smoke weed order much restaurant | social |
| pls sell weed distribute alcohol protest venue maintain peace amp keep aggressionviolence is… | supply disruption |
| order complete drug alcohol diversion program felony sentence day jail for… | supply disruption |
| sell weed literally major felony essential business open pandemic much america amp yet many are… | supply disruption |
| local deli sell mexican coke turn moldy expiration date stay safe | supply disruption |
| say weed come strong probably order least wing good | supply disruption |
| hitchens cancel today would likely often drunk inappropriate young… | medical disruption |
| rehab piss drinker | medical disruption |
| deadly public health crisis year terrify talk potus politica… | medical disruption |
| problem drug alcohol addiction find best rehab facility meet need factor | medical disruption |
| mitch mcconnell cocaine good amp lace fine blow need rehab idiot | medical disruption |

## **2 Result**

### **2.1 Trend Analysis**

Figure S1. Proportion of substance use tweets count from 2019 through 2021


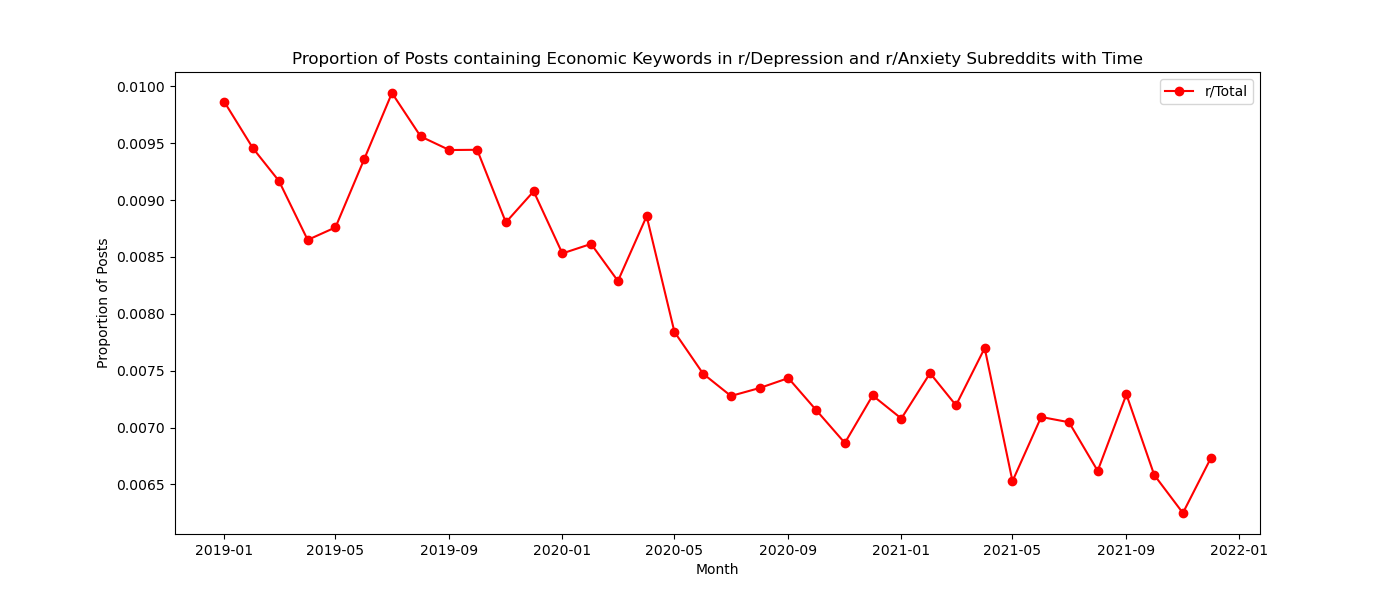


Figure S2. Substance use trend in pre, during and post COVID-19 pandemic
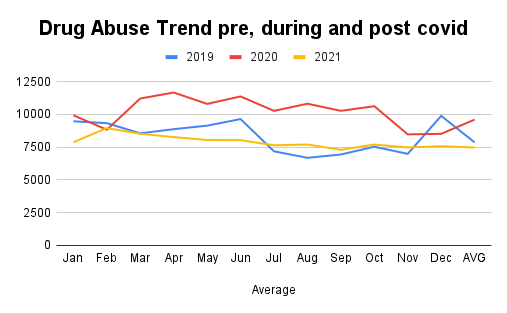


### **2.2 Substance Use Trend in 7 days Before and After the COVID-19 Pandemic declaration day**

Figure S3. Substance use trend in 7 days before and after the COVID-19 pandemic declaration day


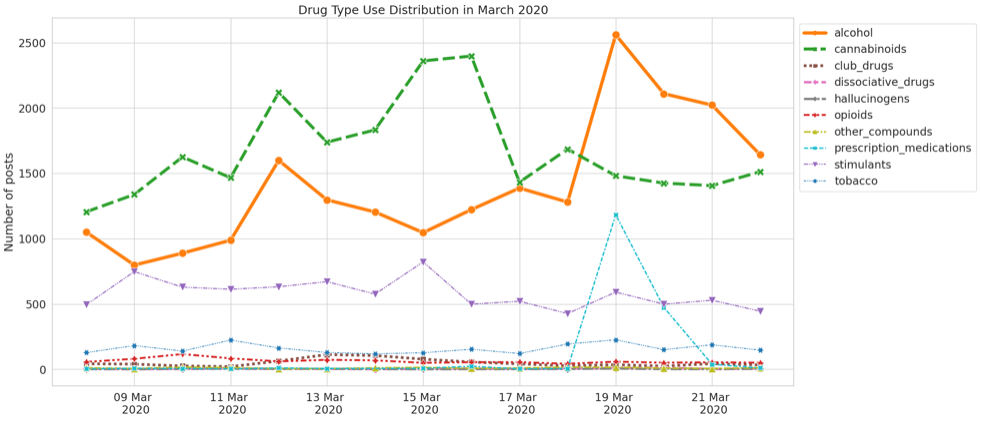


## **3 k-means Clustering Analysis**

### **3.1 k-means Clustering Analysis**

Figure S4. Elbow diagram to predict ‘k’ value in k-mean clustering method.


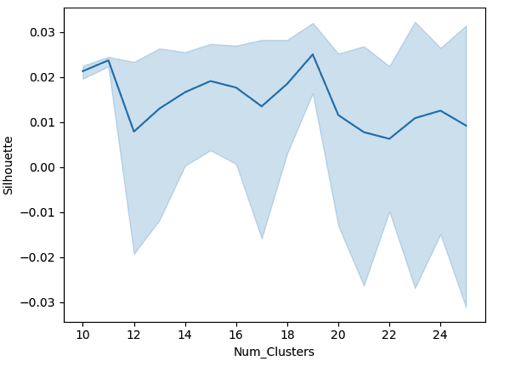


Table S8. k-mean clustering keywords performed for 2020 substance use posts

| Cluster 0: fuck nobody get drink smoke drunk shit weed without cunt say know high need give people nigga take really amp let stop right one make beer day man think even  Cluster 1: weed smoke weed smoke one need day amp people get high make know time think good shit say really man lol smell would buy give love nigga thing see come call  Cluster 2: glass fire lit glass wine wine get lit get liquid wine glass drink one memphis need take look amp smoke water day know see make pour tonight would shit good right night without  Cluster 3: show head hit weed zoom food drink fly folk follow food force foot forever forget four free frequency floor flavor fresh fix feeling felt fight figure fill finally find fine fine wine  Cluster 4: high get high get low high high fly time know take think right one amp people even make say need horse give shit day lol still let look much really school five  Cluster 5: drink get smoke drunk one day know people need say make time amp would think take coffee good let really lol shit eat even never right come still water night  Cluster 6: smoke pop pop smoke get cigarette know say amp get smoke would one nigga weed think need come day smoke cigarette blunt shit lol people smoke weed see let even smoke crack really time crack  Cluster 7: beer hold beer hold drink beer drink get madison madison beer one good day amp make buy would cold say time root craft need love new think today come know bottle great well  Cluster 8: alcoholic asf eligible heroin mercury tree downgrade hide innocence get turn know amp one year make voter people say would vote think fine drink need look even day good time  Cluster 9: everybody drink get smoke know drunk lol weed nobody tonight shit else sleep say need high amp talk right house love time one except good lmao piss stop take make  Cluster 10: smoking stop smoking stop quit cigarette get weed gun smoke say crack blunt shit amp start know think hot good make day one love need time people lol would really fuck  Cluster 11: know tequila upgrade street say drunk slow make amp thing need one people good day think time year covid give look would right shit well really lmfao call trump come  Cluster 12: wine bottle bottle wine drink wine red red wine drink amp get fine fine wine good wine drunk make wineglass age get wine white need night time love taste day one drunk buy know tonight would  Cluster 13: bitch fuck get smoke drunk drink nigga stop need say shit high lmfao know take weed amp call really bad lmao love crack street one let yes tire money coke  Cluster 14: alcohol pain take coke crack drinking drug cigarette liquor cocaine stop nose know say make people need amp blunt one store think use liquor store feel day time good much would  Cluster 15: get tire tired worry hate tire get honestly stop shit one use slowly cheat must already person day figure fire leave bitch honest really half lol everyday even amp bro well  Cluster 16: bar sleep way get drink night drunk need one day take well amp good know eat tonight say really make still think hour smoke people would back time tire right  Cluster 17: drunk get drunk get love night tonight friend know think home say time amp lol right one make shit really miss day people last see tweet take call let look come  Cluster 18: blunt roll hit need smoke smoke blunt get amp right day light pa let fat put one say would nigga lol time think shit first know bitch really drop another good |
| --- |

## **4 Factor Analysis**

### **4.1 Factor Analysis**

Figure S5. Scree plot for identifying the number of factors

**
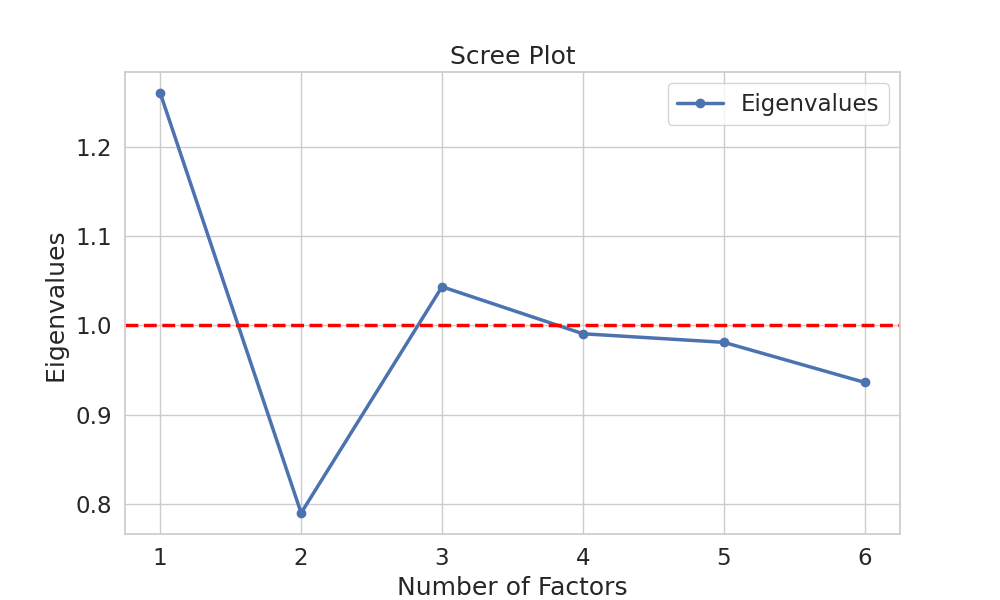
**
